# Clinicopathological and immunological features of new onset kidney disease: a rare event after SARS-CoV-2 vaccination

**DOI:** 10.1093/nsr/nwac034

**Published:** 2023-04-14

**Authors:** Yue-Miao Zhang, Xing-Zi Liu, Zhao Zhang, Tai-Cheng Zhou, Xin Zhang, Hong-Yu Yang, Meng Tan, Nan Hu, Su-Fang Shi, Fang Wang, Rong Xu, Li-Jun Liu, Su-Xia Wang, Gang Liu, Fu-De Zhou, Ming-Hui Zhao, Hong Zhang, Ji-Cheng Lv, Ya-Ping Zhang, Zi-Jie Zhang, Li Yang

**Affiliations:** Renal Division, Department of Medicine, Peking University First Hospital, Beijing 100034, China; Renal Pathology Center, Institute of Nephrology, Peking University, Beijing 100034, China; Key Laboratory of Renal Disease, Ministry of Health of China, Beijing 100034, China; Key Laboratory of CKD Prevention and Treatment, Ministry of Education of China, Beijing 100034, China; Research Units of Diagnosis and Treatment of Immune-Mediated Kidney Diseases, Chinese Academy of Medical Sciences, Beijing 100034, China; State Key Laboratory of Genetic Resources and Evolution, Kunming Institute of Zoology, Chinese Academy of Sciences, Kunming 650223, China; Renal Division, Department of Medicine, Peking University First Hospital, Beijing 100034, China; Renal Pathology Center, Institute of Nephrology, Peking University, Beijing 100034, China; Key Laboratory of Renal Disease, Ministry of Health of China, Beijing 100034, China; Key Laboratory of CKD Prevention and Treatment, Ministry of Education of China, Beijing 100034, China; Research Units of Diagnosis and Treatment of Immune-Mediated Kidney Diseases, Chinese Academy of Medical Sciences, Beijing 100034, China; Renal Division, Department of Medicine, Peking University First Hospital, Beijing 100034, China; Renal Pathology Center, Institute of Nephrology, Peking University, Beijing 100034, China; Key Laboratory of Renal Disease, Ministry of Health of China, Beijing 100034, China; Key Laboratory of CKD Prevention and Treatment, Ministry of Education of China, Beijing 100034, China; Research Units of Diagnosis and Treatment of Immune-Mediated Kidney Diseases, Chinese Academy of Medical Sciences, Beijing 100034, China; Central Laboratory and Liver Disease Research Center, The Affiliated Hospital of Yunnan University, Kunming 650091, China; Renal Division, Department of Medicine, Peking University First Hospital, Beijing 100034, China; Renal Pathology Center, Institute of Nephrology, Peking University, Beijing 100034, China; Key Laboratory of Renal Disease, Ministry of Health of China, Beijing 100034, China; Key Laboratory of CKD Prevention and Treatment, Ministry of Education of China, Beijing 100034, China; Research Units of Diagnosis and Treatment of Immune-Mediated Kidney Diseases, Chinese Academy of Medical Sciences, Beijing 100034, China; Renal Division, Department of Medicine, Peking University First Hospital, Beijing 100034, China; Renal Pathology Center, Institute of Nephrology, Peking University, Beijing 100034, China; Key Laboratory of Renal Disease, Ministry of Health of China, Beijing 100034, China; Key Laboratory of CKD Prevention and Treatment, Ministry of Education of China, Beijing 100034, China; Research Units of Diagnosis and Treatment of Immune-Mediated Kidney Diseases, Chinese Academy of Medical Sciences, Beijing 100034, China; Renal Division, Department of Medicine, Peking University First Hospital, Beijing 100034, China; Renal Pathology Center, Institute of Nephrology, Peking University, Beijing 100034, China; Key Laboratory of Renal Disease, Ministry of Health of China, Beijing 100034, China; Key Laboratory of CKD Prevention and Treatment, Ministry of Education of China, Beijing 100034, China; Research Units of Diagnosis and Treatment of Immune-Mediated Kidney Diseases, Chinese Academy of Medical Sciences, Beijing 100034, China; Renal Division, Department of Medicine, Peking University First Hospital, Beijing 100034, China; Renal Pathology Center, Institute of Nephrology, Peking University, Beijing 100034, China; Key Laboratory of Renal Disease, Ministry of Health of China, Beijing 100034, China; Key Laboratory of CKD Prevention and Treatment, Ministry of Education of China, Beijing 100034, China; Research Units of Diagnosis and Treatment of Immune-Mediated Kidney Diseases, Chinese Academy of Medical Sciences, Beijing 100034, China; Renal Division, Department of Medicine, Peking University First Hospital, Beijing 100034, China; Renal Pathology Center, Institute of Nephrology, Peking University, Beijing 100034, China; Key Laboratory of Renal Disease, Ministry of Health of China, Beijing 100034, China; Key Laboratory of CKD Prevention and Treatment, Ministry of Education of China, Beijing 100034, China; Research Units of Diagnosis and Treatment of Immune-Mediated Kidney Diseases, Chinese Academy of Medical Sciences, Beijing 100034, China; Renal Division, Department of Medicine, Peking University First Hospital, Beijing 100034, China; Renal Pathology Center, Institute of Nephrology, Peking University, Beijing 100034, China; Key Laboratory of Renal Disease, Ministry of Health of China, Beijing 100034, China; Key Laboratory of CKD Prevention and Treatment, Ministry of Education of China, Beijing 100034, China; Research Units of Diagnosis and Treatment of Immune-Mediated Kidney Diseases, Chinese Academy of Medical Sciences, Beijing 100034, China; Renal Division, Department of Medicine, Peking University First Hospital, Beijing 100034, China; Renal Pathology Center, Institute of Nephrology, Peking University, Beijing 100034, China; Key Laboratory of Renal Disease, Ministry of Health of China, Beijing 100034, China; Key Laboratory of CKD Prevention and Treatment, Ministry of Education of China, Beijing 100034, China; Research Units of Diagnosis and Treatment of Immune-Mediated Kidney Diseases, Chinese Academy of Medical Sciences, Beijing 100034, China; Renal Division, Department of Medicine, Peking University First Hospital, Beijing 100034, China; Renal Pathology Center, Institute of Nephrology, Peking University, Beijing 100034, China; Key Laboratory of Renal Disease, Ministry of Health of China, Beijing 100034, China; Key Laboratory of CKD Prevention and Treatment, Ministry of Education of China, Beijing 100034, China; Research Units of Diagnosis and Treatment of Immune-Mediated Kidney Diseases, Chinese Academy of Medical Sciences, Beijing 100034, China; Renal Division, Department of Medicine, Peking University First Hospital, Beijing 100034, China; Renal Pathology Center, Institute of Nephrology, Peking University, Beijing 100034, China; Key Laboratory of Renal Disease, Ministry of Health of China, Beijing 100034, China; Key Laboratory of CKD Prevention and Treatment, Ministry of Education of China, Beijing 100034, China; Research Units of Diagnosis and Treatment of Immune-Mediated Kidney Diseases, Chinese Academy of Medical Sciences, Beijing 100034, China; Laboratory of Electron Microscopy, Pathological Centre, Peking University First Hospital, Beijing 100034, China; Renal Division, Department of Medicine, Peking University First Hospital, Beijing 100034, China; Renal Pathology Center, Institute of Nephrology, Peking University, Beijing 100034, China; Key Laboratory of Renal Disease, Ministry of Health of China, Beijing 100034, China; Key Laboratory of CKD Prevention and Treatment, Ministry of Education of China, Beijing 100034, China; Research Units of Diagnosis and Treatment of Immune-Mediated Kidney Diseases, Chinese Academy of Medical Sciences, Beijing 100034, China; Renal Division, Department of Medicine, Peking University First Hospital, Beijing 100034, China; Renal Pathology Center, Institute of Nephrology, Peking University, Beijing 100034, China; Key Laboratory of Renal Disease, Ministry of Health of China, Beijing 100034, China; Key Laboratory of CKD Prevention and Treatment, Ministry of Education of China, Beijing 100034, China; Research Units of Diagnosis and Treatment of Immune-Mediated Kidney Diseases, Chinese Academy of Medical Sciences, Beijing 100034, China; Renal Division, Department of Medicine, Peking University First Hospital, Beijing 100034, China; Renal Pathology Center, Institute of Nephrology, Peking University, Beijing 100034, China; Key Laboratory of Renal Disease, Ministry of Health of China, Beijing 100034, China; Key Laboratory of CKD Prevention and Treatment, Ministry of Education of China, Beijing 100034, China; Research Units of Diagnosis and Treatment of Immune-Mediated Kidney Diseases, Chinese Academy of Medical Sciences, Beijing 100034, China; Renal Division, Department of Medicine, Peking University First Hospital, Beijing 100034, China; Renal Pathology Center, Institute of Nephrology, Peking University, Beijing 100034, China; Key Laboratory of Renal Disease, Ministry of Health of China, Beijing 100034, China; Key Laboratory of CKD Prevention and Treatment, Ministry of Education of China, Beijing 100034, China; Research Units of Diagnosis and Treatment of Immune-Mediated Kidney Diseases, Chinese Academy of Medical Sciences, Beijing 100034, China; Renal Division, Department of Medicine, Peking University First Hospital, Beijing 100034, China; Renal Pathology Center, Institute of Nephrology, Peking University, Beijing 100034, China; Key Laboratory of Renal Disease, Ministry of Health of China, Beijing 100034, China; Key Laboratory of CKD Prevention and Treatment, Ministry of Education of China, Beijing 100034, China; Research Units of Diagnosis and Treatment of Immune-Mediated Kidney Diseases, Chinese Academy of Medical Sciences, Beijing 100034, China; State Key Laboratory of Genetic Resources and Evolution, Kunming Institute of Zoology, Chinese Academy of Sciences, Kunming 650223, China; State Key Laboratory for Conservation and Utilization of Bio-Resource and School of Life Sciences, Yunnan University, Kunming 650091, China; Renal Division, Department of Medicine, Peking University First Hospital, Beijing 100034, China; Renal Pathology Center, Institute of Nephrology, Peking University, Beijing 100034, China; Key Laboratory of Renal Disease, Ministry of Health of China, Beijing 100034, China; Key Laboratory of CKD Prevention and Treatment, Ministry of Education of China, Beijing 100034, China; Research Units of Diagnosis and Treatment of Immune-Mediated Kidney Diseases, Chinese Academy of Medical Sciences, Beijing 100034, China

**Keywords:** kidney disease, SARS-CoV-2, vaccination, abnormal immune response

## Abstract

The onset of various kidney diseases has been reported after severe acute respiratory syndrome coronavirus 2 (SARS-CoV-2) vaccination. However, detailed clinical and pathological features are lacking. We screened and analyzed patients with newly diagnosed kidney diseases after inactivated SARS-CoV-2 vaccination in Peking University First Hospital from January 2021 to August 2021, and compared them with the reported cases in the literature. We obtained samples of blood, urine and renal biopsy tissues. Clinical and laboratory information, as well as light microscopy, immunostaining and ultrastructural observations, were described. The SARS-CoV-2 spike protein and nucleoprotein were stained using the immunofluorescence technique in the kidney biopsy samples. SARS-CoV-2 specific antibodies were tested using magnetic particle chemiluminescence immunoassay. The study group included 17 patients with a range of conditions including immune-complex-mediated kidney diseases (IgA nephropathy, membranous nephropathy and lupus nephritis), podocytopathy (minimal change disease and focal segmental glomerulosclerosis) and others (antineutrophil-cytoplasmic-antibody-associated vasculitis, anti-glomerular basement membrane nephritis, acute tubulointerstitial nephritis and thrombotic microangiopathy). Seven patients (41.18%) developed renal disease after the first dose and ten (58.82%) after the second dose. The kidney disease spectrum as well as clinicopathological features are similar across different types of SARS-CoV-2 vaccines. We found no definitive evidence of SARS-CoV-2 spike protein or nucleoprotein deposition in the kidney biopsy samples. Seropositive markers implicated abnormal immune responses in predisposed individuals. Treatment and follow-up (median = 86 days) showed that biopsy diagnosis informed treatment and prognosis in all patients. In conclusion, we observed various kidney diseases following SARS-CoV-2 vaccine administration, which show a high consistency across different types of SARS-CoV-2 vaccines. Our findings provide evidence against direct vaccine protein deposition as the major pathomechanism, but implicate abnormal immune responses in predisposed individuals. These findings expand our understanding of SARS-CoV-2 vaccine renal safety.

## INTRODUCTION

Coronavirus disease 2019 (COVID-19), caused by severe acute respiratory syndrome coronavirus 2 (SARS-CoV-2), has been reported to cause kidney injury through a variety of mechanisms, such as direct interaction with its cellular receptor angiotensin-converting enzyme 2 (ACE2), which is widely expressed in proximal tubular cells as well as podocytes [1–4] and aberrant host immune responses [5]. In turn, kidney injury is linked to severe disease progression and increased death risk in hospitalized patients with COVID-19 [6–9]. In the absence of disease-specific drugs, vaccines against SARS-CoV-2, which have been reported to be effective and safe by large clinical trials [10–12], represent the most promising way to protect these vulnerable populations. However, these trials might be less effective at identifying long-term or rare, but significant, side effects such as kidney diseases [13].

The colossal impact of COVID-19 is driving the largest vaccination campaign in human history. With billions of doses of vaccines administered worldwide against COVID-19, isolated reports of both *de novo* and recurrent kidney diseases are emerging, including IgA nephropathy (IgAN), minimal change disease (MCD), membranous nephropathy (MN), acute kidney injury (AKI) and even acute allograft rejection [14]. Until March 2022, kidney involvement was largely described as being associated with the novel mRNA-based vaccines (summarized in Table 1 and see more details in Supplementary Table 1). With regard to the other four types of SARS-CoV-2 vaccine, billions of doses of these inactivated vaccines have been administered around the world, and there was one case report of the relapse of MN after receiving the inactivated SARS-CoV-2 vaccine (CoronaVac) [15]. As far, these reports mainly focused on describing the clinicopathological information while the immunological features as well as long-term prognosis remain to be explored.

**Table 1.** New onset or relapsing kidney diseases after SARS-CoV-2 vaccination in the literature till March 2022.

	Vaccine types			
Disease types	mRNA vaccine (*n* = 60)	Adenovirus vaccine (*n* = 17)	Inactivated vaccine (*n* = 2)	Total (*n* = 79)
IgAN/IgAV	30 (50.00%)	1 (5.88%)	-	31 (39.24%)
GN vasculitis	-	1 (5.88%)	-	1 (1.27%)
MCD	14 (23.33%)	7 (41.18%)	-	21 (26.58%)
AAV	5 (8.33%)	3 (17.65%)	-	8 (10.13%)
LN	2 (3.33%)	2 (11.76%)	-	4 (5.06%)
MN	2 (3.33%)	-	1 (50.00%)	3 (3.80%)
AIN	2 (3.33%)	-	1 (50.00%)	3 (3.80%)
anti-GBM GN	2 (3.33%)	-	-	2 (2.53%)
Collapsing GN	-	2 (11.76%)	-	2 (2.53%)
FSGS	1 (1.67%)	-	-	1 (1.27%)
TMA	-	1 (5.88%)	-	1 (1.27%)
IgG4RD	1 (1.67%)	-	-	1 (1.27%)
Acute rejection	1 (1.67%)	-	-	1 (1.27%)

Abbreviations: AAV, anti-neutrophil cytoplasmic antibody (ANCA)-associated vasculitis; AIN, acute interstitial nephritis; FSGS, focal segmental glomerulosclerosis; GBM, glomerular basement membrane; GN, glomerulonephritis; IgAN, IgA nephropathy; IgAV, IgA vasculitis; IgG4RD, IgG4 related disease; LN, lupus nephritis; MCD, minimal change disease; MN, membranous glomerulopathy; TMA, thrombotic microangiopathy.

More importantly, multiple platform vaccines against SARS-CoV-2 have now been authorized for use in various countries. Inactivated and mRNA vaccines, the two most widely deployed vaccines globally, as examples, inactivated SARS-CoV-2 vaccines are heat- or chemically inactivated SARS-CoV-2 [16], while the mRNA vaccine delivers SARS-CoV-2 spike-protein-encoding mRNA via lipid nanoparticles. Although both of them have been shown to be effective and safe on the prevention of COVID-19 [10–12], mRNA vaccines were suggested to induce stronger balanced humoral and T cell immunity [17], while inactivated vaccines elicit less adverse reactions. Moreover, while mRNA vaccines are reported to stimulate Immunoglobulin (Ig) G, IgA and IgM responses, inactivated vaccines seem to mainly stimulate IgG and IgM, but not IgA, responses [18]. Thus, it would be also interesting to ask whether the clinicopathological features of postvaccination kidney diseases differ by vaccine types.

In this study, we systemically review the reported cases in the literature, and provide a kidney biopsy series of patients who were admitted to Peking University First Hospital, a large renal center in North China, with new-onset kidney diseases after receiving SARS-CoV-2 vaccines.

## RESULTS

### Baseline information

We screened the hospitalized patients admitted to renal department of Peking University First Hospital between 1 January 2021 and 10 August 2021. After SARS-CoV-2 vaccination, 20 patients presented with kidney abnormalities within one month. Among them, we excluded three patients: one receiving a non-inactivated vaccine, one showing spontaneous remission disease course and one using a nephrotoxic drug right before disease onset. Finally, we included 17 patients, distributing in 13 prefecture-level cities or districts in North China, for analysis: 9 (52.94%) were female and 8 (47.06%) were male. As shown in Table 2, their median age was 39 years (interquartile range (IQR), 33–62.5) and the median intervals between vaccination and blood sampling were 36.5 days and 44.5 days after the first and second dose of vaccination, respectively. Out of the 17 patients, 4 did not have a significant medical history and the other 13 patients had one or more comorbidities, including 5 with hypertension, 3 with proteinuria (but no previous kidney biopsy), 1 with a fatty liver, 1 with gout, 1 with asthma, 1 with rhinitis, 1 with gall stones, 1 with arthrodynia, 1 with diabetes, 1 with obesity, 1 with psoriasis, 1 with gastroesophageal reflux disease, 1 with cataracts and 1 with pruritus. Due to disease severity, five of them received immunosuppressive therapy before admission. The detailed clinical and laboratory data of these 17 patients are summarized in Supplementary Table 2. Interestingly, the demographic information and comorbidities of the kidney disease patients after SARS-CoV-2 vaccination were comparable across different types of vaccines, except for elevated age of patients in the adenoviral vectored vaccine-associated patients (Supplementary Fig. 1). In addition, we enrolled 16 volunteers who received inactivated SARS-CoV-2 vaccines but showed no significant adverse effects as controls. Their median time of blood sampling was 30 days and 37 days after the first dose and the second dose of vaccination, respectively. We also included pathology-matched kidney disease patients (*n* = 14) and healthy individuals (*n* = 40) recruited in 2019 (before the emergence of COVID-19) as controls to rule out potential cross reactivities.

**Table 2.** Baseline information of the participants in this study.

	With vaccination		Without vaccination	
Variables	New-onset kidney disease patients (*n* = 17)	Participants showed no adverse effects (*n* = 16)	Pathology-matched kidney disease patients (*n* = 14)	Healthy controls (*n* = 40)
Age, years, median (IQR^a^)	39 (33–62.5)	38 (29–53)	58.5 (33.5–63.5)	32 (24.5–48)
Sex, *n* (%)				
Male	8 (47)	6 (37.5)	7 (50)	14 (35)
Female	9 (53)	10 (62.5)	7 (50)	26 (65)
Comorbidities, *n* (%)				
Proteinuria	3 (18)	0 (0)	9 (64)	0 (0)
Hypertension	5 (29)	0 (0)	4 (29)	0 (0)
Diabetes	1 (6)	0 (0)	2 (14)	0 (0)
Obesity	1 (6)	0 (0)	0 (0)	0 (0)
Cataracts	1 (6)	0 (0)	0 (0)	0 (0)
Fatty liver	1 (6)	0 (0)	0 (0)	0 (0)
Gall stone	1 (6)	0 (0)	0 (0)	0 (0)
Gastrointestinal disease	1 (6)	0 (0)	2 (14)	0 (0)
Asthma	1 (6)	0 (0)	0 (0)	0 (0)
Rhinitis	1 (6)	0 (0)	0 (0)	0 (0)
Arthrodynia	1 (6)	0 (0)	0 (0)	0 (0)
Psoriasis	1 (6)	0 (0)	0 (0)	0 (0)
Gout	1 (6)	0 (0)	0 (0)	0 (0)
Pruritus	1 (6)	0 (0)	0 (0)	0 (0)
Cardiovascular disease	0 (0)	0 (0)	2 (14)	0 (0)
IS drugs before blood sampling, *n* (%)	5 (29)	0 (0)	1 (7.14)	0 (0)
Sampling^b^, *n*, days, median (IQR)				
After the first dose	4, 36.5 (29–149)	4, 30 (27–36)	N/A	N/A
After the second dose	12, 44.5 (33–74)	12, 27 (25–28)	N/A	N/A

^a^Abbreviations: IQR, interquartile range; IS, immunosuppressive; N/A, not applicable. ^b^Blood samples were available in 16 of the 17 patients.

### Phenotypes and timeline

**Figure 1.**
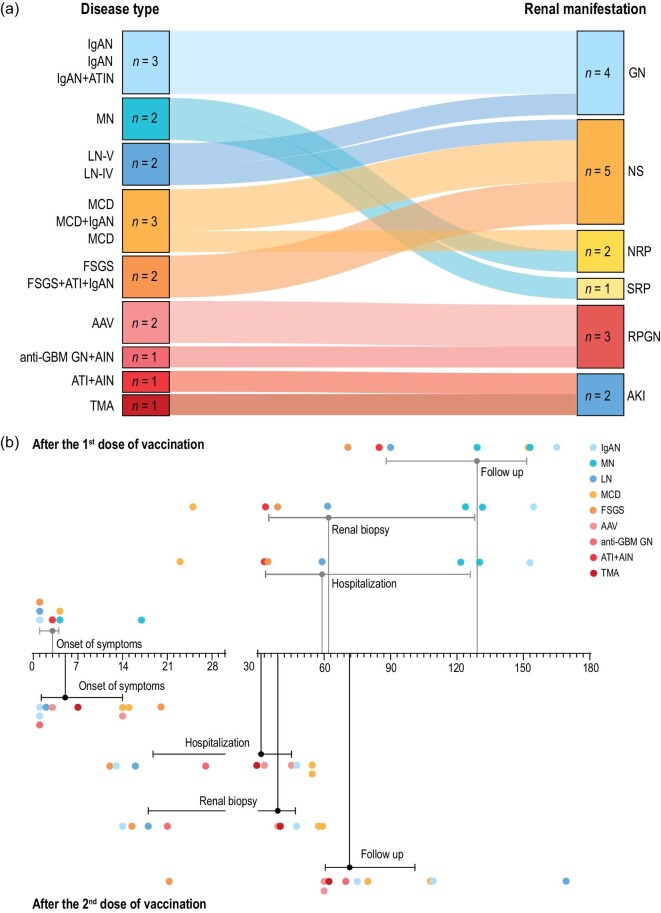
Distribution of phenotypes, initial manifestations and timeline of the 17 new onset kidney disease patients after inactivated SARS-CoV-2 vaccination. Abbreviations: AAV, anti-neutrophil cytoplasmic antibody (ANCA)-associated vasculitis; AKI, acute kidney injury; AIN, acute interstitial nephritis; ATI, acute tubular injury; ATIN, acute tubular-interstitial nephropathy; FSGS, focal segmental glomerulosclerosis; GBM, glomerular basement membrane; GN, glomerulonephritis; IgAN, IgA nephropathy; LN, lupus nephritis; MCD, minimal change disease; MN, membranous nephropathy; NRP, nephrotic range proteinuria; NS, nephrotic syndrome; RPGN, rapidly progressive glomerulonephritis; SRP, sub-nephrotic range proteinuria; TMA, thrombotic microangiopathy.

We observed a wide spectrum of kidney diseases, almost all of which were immune-mediated kidney diseases. Among them, immune-complex-mediated kidney diseases included three cases with IgAN, two with MN and two with lupus nephritis (LN); podocytopathy included three cases with MCD and two cases with focal segmental glomerulosclerosis (FSGS). The others included two cases with antineutrophil cytoplasmic antibody (ANCA)-associated vasculitis (AAV), one with anti-glomerular basement membrane (GBM) glomerulonephritis (anti-GBM GN), one with acute tubulointerstitial nephritis (ATIN) and one with thrombotic microangiopathy (TMA) (Fig. 1a). We observed a highly consistent kidney disease spectrum across inactivated, mRNA, and adenoviral vectored vaccines, indicating the potential shared immunological features of kidney disease after SARS-CoV-2 vaccination (Supplementary Fig. 2a). Our 17 patients presented with typical initial manifestations. Specifically, immune-complex-mediated kidney diseases mainly presented with glomerulonephritis, podocytopathy with nephrotic syndrome, AAV and anti-GBM GN with rapidly progressive glomerulonephritis (RPGN), and ATIN and TMA with AKI. Notably, both patients diagnosed with LN had autoimmune antibodies and showed systemic manifestations such as joint swelling. They met the 2019 European League Against Rheumatism/American College of Rheumatology classification criteria for systemic lupus erythematosus [19]. Renal biopsy revealed LN class IV and class V respectively (Supplementary Table 2).

The timelines of disease onset, hospital admission, diagnosis and follow-up are shown in Fig. 1b. Similar with the mRNA vaccines, seven patients (7/17, 41.18%) developed symptoms following the first dose of vaccination and 10 patients (10/17, 58.82%) after the second dose. Conversely, more patients developed kidney disease after the first dose of adenoviral vectored vaccines (Supplementary Fig. 2b and 2c). The median time of disease onset was 3 (IQR, 1–4) days after the first dose, while it was relatively longer at 5 (IQR, 1.25–14) days after the second dose. Overall, compared to the first dose, patients developing kidney disease after the second dose of vaccination showed higher levels of disease severity, which might lead to their relatively earlier hospitalization. The median time of admission, diagnosis and follow-up after the first dose was 59 (IQR, 33.5–126) days, 62 (IQR, 35–128) days and 129 (IQR, 88–151.5) days, respectively. It was 31.5 (IQR, 18.75–45.25) days, 39 (IQR, 18–47) days and 71.5 (IQR, 60.5–101) days after the second dose.

### Clinicopathological features

**Figure 2.**
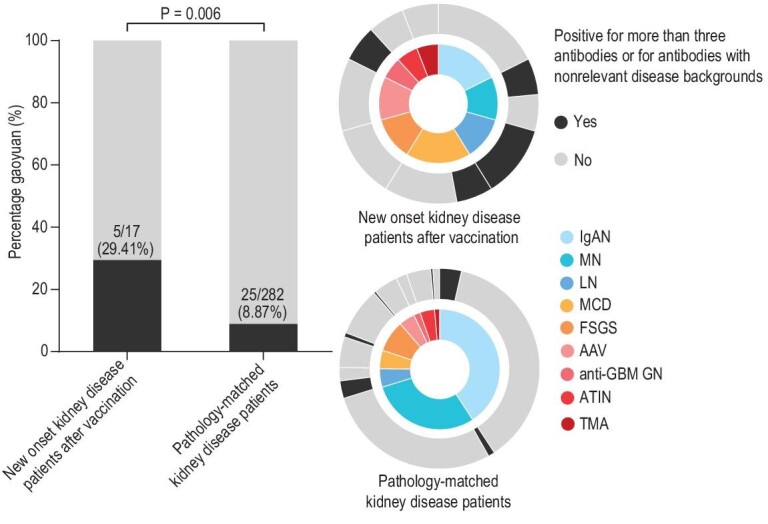
Comparison of serological data of the 17 patients with new-onset kidney disease after inactivated SARS-CoV-2 vaccination and pathology-matched kidney disease controls enrolled in 2019. Abbreviations: AAV, anti-neutrophil cytoplasmic antibody (ANCA)-associated vasculitis; ATIN, acute tubular-interstitial nephropathy; FSGS, focal segmental glomerulosclerosis; GBM, glomerular basement membrane; GN, glomerulonephritis; IgAN, IgA nephropathy; LN, lupus nephritis; MCD, minimal change disease; MN, membranous nephropathy; SARS-CoV-2, severe acute respiratory syndrome coronavirus 2; TMA, thrombotic microangiopathy.

At presentation, the study group had a median serum creatinine of 118.37 μmol/L (range, 58.4–546 μmol/L), median urine protein of 4.32 g/24 h and median serum albumin of 31.5 g/L. Eleven patients had microhematuria (more than three red blood cells per high-power field). There was no significant abnormality of peripheral complete blood counts. In addition, we also observed the increased levels of IgA and the decreased levels of complement C3 or C4 in four and two patients, respectively. Positive serologies included five patients with antinuclear antibodies (including one with anti-double-stranded DNA antibodies), two with ANCA, one with anti-Jo-1 antibodies, one with anti-histone (HIS), anti-nucleosome and anti-proliferating cell nuclear antigen (PCNA) antibodies, two with anti-Sjogren's-syndrome-related antigen A (SS-A) antibodies, one with anti-ribosomal ribonucleoprotein (rRNP) antibodies, one with anti-GBM antibodies and one with anti-M-type phospholipase A2 receptor (PLA2R) antibodies (Table 3). Notably, five of these patients were positive for three types of antibodies or showed antibodies with non-relevant disease backgrounds. This proportion was significantly higher than that in an independent cohort (*n* = 282) included from 1 January 2019 to 30 April 2019 (Supplementary Table 3), suggesting an aggressive humoral immune response in these patients (29.41% vs. 8.87%, Fig. 2).

**Table 3.** Serological data of the 17 patients with new-onset kidney disease after inactivated SARS-CoV-2 vaccination.

ID	Diagnosis	ANA	dsDNA	Jo-1	HIS	ANuA	PCNA	SS-A	rRNP	MPO-ANCA	Anti-GBM	PLA2R
1	IgAN	-	-	-	-	-	-	-	-	-	-	-
2	IgAN	-	-	-	-	-	-	-	-	-	-	-
3	IgAN + ATIN	-	-	-	-	-	-	-	-	-	-	-
4	MCD	1 : 100↑	-	39↑	-	-	-	-	-	-	-	-
5	MCD + IgAN	-	-	-	-	-	-	-	-	-	-	-
6	MCD	-	-	-	-	-	-	-	-	-	-	-
7	AAV^a^	-	-	-	-	-	-	-	-	494.50↑	-	-
8	AAV	-	-	-	-	-	-	-	-	>200↑	-	-
9	FSGS + ATI + IgAN	-	-	-	-	-	-	-	-	-	-	-
10	FSGS	-	-	-	-	-	-	-	-	-	-	-
11	SLE, LN-IV	1 : 10 000↑	791↑	-	42↑	30↑	29↑	-	-	-	-	-
12	SLE, LN-V	1 : 1000↑	-	-	-	-	-	155↑	149↑	-	-	-
13	MN	-	-	-	-	-	-	-	-	-	-	-
14	MN	1 : 3200↑	-	-	-	-	-	113↑	-	-	-	-
15	ATI + AIN	-	-	-	-	-	-	-	-	-	-	-
16	anti-GBM GN + AIN	-	-	-	-	-	-	-	-	-	123↑	37↑
17	TMA	1 : 100↑	-	-	-	-	-	-	-	-	-	-

Note: Antibody test includes anti-nuclear antibodies (ANA) (1:<100), anti-double-stranded DNA (dsDNA) antibodies (<100 IU/mL), anti-nuclear ribonucleoprotein (nRNP) antibodies (<25), anti-Sm antibodies (<25), anti-Sjogren's-syndrome-related antigen A (SS-A) antibodies (<25), anti-Sjogren's-syndrome-related antigen B (SS-B) antibodies (<25), anti-Scl-70 antibodies (<25), anti-Jo-1 antibodies (<25), anti-ribosomal ribonucleoprotein (rRNP) antibodies, anti-mitochondrial M2 antibodies (AMA-M2) (<25), anti-histone (HIS) antibodies (<25), anti-nucleosome antibody (ANuA) (<25), anti-centromere protein (CENP) B antibodies (<25), anti-proliferating cell nuclear antigen (PCNA) antibodies (<25), anti-neutrophil cytoplasmic antibody (ANCA) (negative), ANCA specific for proteinase 3 (PR3-ANCA) (<20), ANCA specific for myeloperoxidase (MPO-ANCA) (<20), anti-glomerular basement membrane (GBM) antibodies (<20) and anti-M-type phospholipase A2 receptor (PLA2R) antibodies (<20 RU/mL). Only the antibodies with at least one positive result were shown in the table and ‘-’ means negative.

^a^Abbreviations: AAV, ANCA-associated vasculitis; AIN, acute interstitial nephritis; ATI, acute tubular injury; ATIN, acute tubular-interstitial nephropathy; FSGS, focal segmental glomerulosclerosis; GBM, glomerular basement membrane; GN, glomerulonephritis; ID, identification number; IgAN, IgA nephropathy; LN, lupus nephritis; MCD, minimal change disease; MN, membranous nephropathy; PLA2R, phospholipase A2 receptor; SARS-CoV-2, severe acute respiratory syndrome coronavirus 2; SLE, systemic lupus erythematosus; TMA, thrombotic microangiopathy.

Among the 17 patients with new-onset kidney disease after vaccination, 14 patients underwent native kidney biopsy during hospitalization, two patients received kidney biopsy before transferring to our hospital and one patient with AAV did not receive kidney biopsy due to advanced age (85 years) and disease severity (requiring dialysis). Consistent with the previous reports of kidney disease after mRNA and adenoviral vectored vaccines (Supplementary Table 1), we observed typical pathological manifestations of these patients such as the main deposition of IgA in the glomerular mesangium of the three cases with IgAN and the positive tissue staining for PLA2R of two cases with MN. Notably, we found 4/16 cases presented with composite pathological diagnoses, including one case with IgAN and one case with anti-GBM GN accompanied by ATI, one case with MCD accompanied by mild IgAN (Oxford Classification of M0E0S0T0C0), and one case with FSGS accompanied by ATI and mild IgAN (Oxford Classification of M0E0S0T0C0). In addition, under light microscopy, acute lesions (i.e. hyper-cellularity, crescents or interstitial inflammation) and chronic lesions (i.e. glomerular sclerosis, tubular atrophy and interstitial fibrosis or vascular sclerosis) co-occurred in 15/16 patients (93.75%), suggesting the susceptibility to kidney disease of these patients. Ultrastructural examination demonstrated dense deposits in 10 out of 14 cases (71.43%) and segmental or diffuse foot process effacement in 13 out of 14 cases (92.86%) (see more details in Supplementary Table 4).

### Immunological features

All 17 patients with new-onset kidney disease after vaccination denied a prior history of SARS-CoV-2 infection. A nasopharyngeal SARS-CoV-2 polymerase chain reaction (PCR) test was conducted in each of the patients at the time of admission, and the results were all negative. SARS-CoV-2 was reported to have renal tropism; therefore, we first stained for the SARS-CoV-2 spike protein and nucleoprotein in renal biopsy samples using the immunofluorescence technique. Staining for SARS-CoV-2 spike protein and nucleoprotein was negative in all cases, in both patients with new-onset kidney disease after vaccination and patients with pathology-matched kidney disease without vaccination (Supplementary Figs 3 and 4). Positive and negative controls of mouse lung tissues showed the expected reactivity of the antibodies. Consistent with this, the levels of SARS-CoV-2-specific IgG, IgA and IgM antibodies were mainly under the cut-off value of 1 S/CO in urine samples (Supplementary Fig. 5), suggesting that the vaccine proteins were unlikely to be deposited in renal tissue.

**Figure 3.**
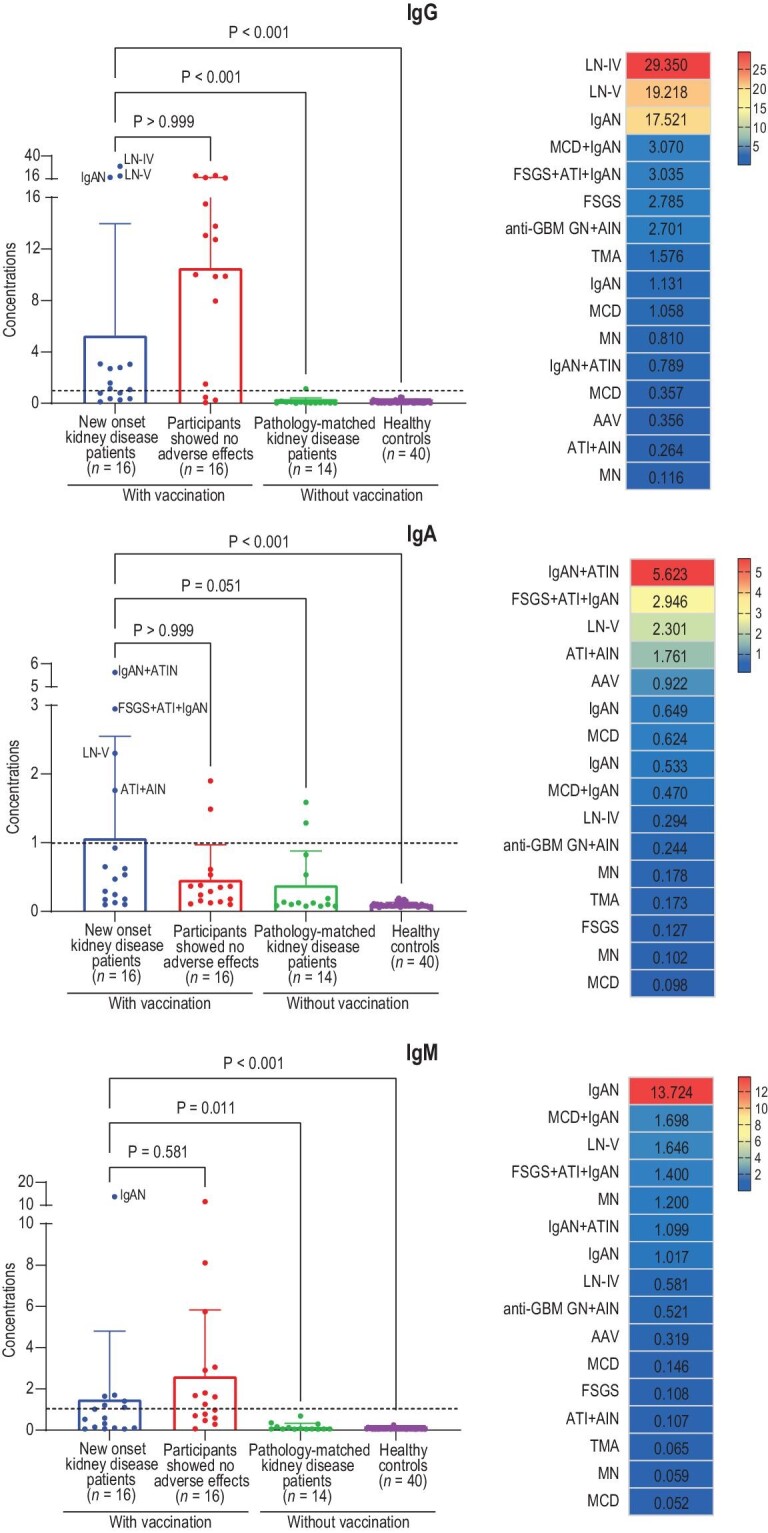
Analyses of the SARS-CoV-2-specific antibodies in serum samples of the new onset kidney disease patients after inactivated SARS-CoV-2 vaccination and controls. Note: the x-axis represents the groups and the y-axis represents the measured chemiluminescence values divided by the cut-off (S/CO). Seroconversion cut-off was defined as 1 S/CO. Abbreviations: AAV, anti-neutrophil cytoplasmic antibody (ANCA)-associated vasculitis; AIN, acute interstitial nephritis; ATI, acute tubular injury; ATIN, acute tubular-interstitial nephropathy; FSGS, focal segmental glomerulosclerosis; GBM, glomerular basement membrane; GN, glomerulonephritis; IgAN, IgA nephropathy; LN, lupus nephritis; MCD, minimal change disease; MN, membranous nephropathy; SARS-CoV-2, severe acute respiratory syndrome coronavirus 2; TMA, thrombotic microangiopathy.

We measured the SARS-CoV-2-specific antibodies after a median interval of 36.5 days after the first dose and after a median interval of 44.5 days after the second dose, respectively (Table 2). After vaccination, serological testing showed seropositive for SARS-CoV-2-specific IgG and IgM antibodies, but not IgA antibodies. Additionally, the levels of SARS-CoV-2-specific IgG and IgM in the new-onset kidney disease patients after vaccination were significantly higher than in controls without vaccination, while it was comparable with people showing no adverse effects after vaccination. Interestingly, we observed stronger IgG, IgA or IgM responses to SARS-CoV-2 for immune-complex-mediated glomerular diseases. For example, the two patients who were diagnosed with new-onset LN, a typical autoimmune disease, after vaccination showed the highest levels of SARS-CoV-2-specific IgG (Fig. 3). In addition, while inactivated vaccines mainly stimulate IgG and IgM, but not IgA, responses, patients with IgAN showed higher levels of SARS-CoV-2-specific IgA (Fig. 3). We further measured the galactose-deficient IgA1 (the key pathogenic feature in IgAN [20]) in the new-onset IgAN patients after vaccination and found it higher than in sex- and age-matched IgAN patients and healthy controls enrolled before 2019 (Supplementary Fig. 6a). We also observed higher levels of autoantibodies (the key pathogenic antibodies in lupus) than in pathology-matched patients with LN enrolled before 2019 (Supplementary Fig. 6b and c). Our findings with regard to the immune response to SARS-CoV-2, together with the absence of direct SARS-CoV-2 antigen deposition in the kidney (Supplementary Figs 3 and 4), highlight the potential for vaccine-induced innate or adaptive immune response abnormalities that in turn trigger the onset of kidney diseases in predisposed individuals.

### Treatment and progression

**Figure 4.**
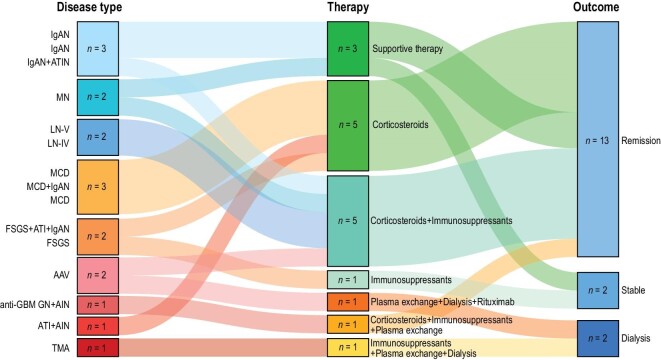
Treatment and prognosis of the 17 new onset kidney disease patients after inactivated SARS-CoV-2 vaccination. Note: the outcomes ‘remission’, ‘stable’ and ‘dialysis’ were defined by improvements of renal function or reductions of pathogenic biomarkers (see details in Supplementary Table 5). Abbreviations: AAV, anti-neutrophil cytoplasmic antibody (ANCA)-associated vasculitis; AIN, acute interstitial nephritis; ATI, acute tubular injury; ATIN, acute tubular-interstitial nephropathy; FSGS, focal segmental glomerulosclerosis; GBM, glomerular basement membrane; GN, glomerulonephritis; IgAN, IgA nephropathy; LN, lupus nephritis; MCD, minimal change disease; MN, membranous nephropathy; TMA, thrombotic microangiopathy.

As shown in Fig. 4, the kidney biopsy findings informed treatment and prognosis. Briefly, supportive therapy and corticosteroids were the main therapy for patients with IgAN and podocytopathy (FSGS and MCD), respectively. For more severe patients presenting with RPGN (AAV, anti-GBM GN, TMA), advanced therapy, including plasma exchange or dialysis, were needed. A majority of the vaccine-induced nephropathy patients (15/17, 88.24%) showed a reversable disease course or had kidney function that remained stable. At the last follow-up, their median serum creatinine had decreased from 118.37 to 102.85 μmol/L (range, 56–641.48 μmol/L); this included seven patients with a decrease in serum creatinine and two who remained dialysis dependent. Among the seven patients showing nephrotic-range proteinuria, there were three with MCD, two with FSGS, one with LN (class IV) and one with MN. A repeat urine protein level was available in five, and all of them had reductions in proteinuria. Repeated testing showed decreased immune markers in the four patients with available data (Supplementary Table 5).

## DISCUSSION

Given the unprecedented worldwide vaccination campaign, isolated cases of kidney disease after vaccination are emerging (mainly in relation to mRNA vaccines). To the best of our knowledge, this is the first prospective study using a biopsy-based series. We also systematically reviewed the reported cases of kidney diseases after vaccination in the literature for comparison. The results advance our knowledge in at least three aspects. First, we observed a wide spectrum of kidney diseases after SARS-CoV-2 vaccination, including IgAN, MN, LN, MCD, FSGS, AAV, anti-GBM GN, ATIN and TMA. The disease spectrum as well as clinicopathological features are similar across different types of vaccines, suggesting potential shared underlying immunological features. Second, we found no definitive evidence of SARS-CoV-2 spike protein or nucleoprotein in the samples using *in situ* immunofluorescent staining, which is an argument against direct vaccine protein deposition as the major pathomechanism. Instead, the high positive rate of multiple or non-relevant antibodies as well as the strong immune response to SARS-CoV-2 in immune-complex-mediated IgAN and LN implicate abnormal immune responses in predisposed individuals. Third, the kidney biopsy findings informed treatment and prognosis, and most of the patients showed a treatable disease course. The median admission delay and median diagnostic delay were relatively long for these patients, suggesting that early diagnosis and treatment would further improve prognosis.

Previously, a wide spectrum of glomerular diseases occurring after SARS-CoV-2 infection were reported [5]. The absence of SARS-CoV-2 in kidney cells of these patients implicated cytokine-mediated effects or aberrant immune responses to virus proteins in predisposed individuals. Thus, even though the risk remains significantly lower than natural SARS-CoV-2 infection, we can reasonably assume that vaccines, containing SARS-CoV-2 antigens, might also have the potential to ‘unmask’ these immune-mediated kidney diseases. Rare, albeit severe, newly diagnosed as well as relapsing glomerular diseases following mRNA SARS-CoV-2 vaccinations have been reported [14], including IgAN, MCD, AAV, ATI and anti-GBM GN. Interestingly, in this study we observed a wider spectrum of kidney injury after SARS-CoV-2 vaccination. These findings enlarge the literature on the setting of inactivated vaccines. Furthermore, the newly observed FSGS, LN and MN have greatly improved our understanding of the whole disease spectrum, which should receive more attention in those patients receiving mRNA vaccines. In fact, the *de novo* MN as well as LN flares after mRNA vaccination were reported subsequently [21–23]. In addition, compared with mRNA vaccines, more patients developed kidney disease after the first dose of an inactivated SARS-CoV-2 vaccination (Supplementary Table 1). The median times at which these patients presented with symptoms were 3 (IQR, 1–4) and 5 (IQR, 1.25–14) days after the first and second dose of the vaccination, respectively. It is recommended that nephrologists closely follow up patients receiving inactivated SARS-CoV-2 vaccines, especially in the mentioned time window.

Although the exact pathogenesis remains to be determined, the wide, but similar spectrum of kidney diseases after SARS-CoV-2 infection, mRNA vaccination and inactivated vaccination suggests the role of direct deposition of SARS-CoV-2 proteins or aberrant immune responses to them, or both (Supplementary Table 6). For example, SARS-CoV-2 might infect the kidney directly via ACE2, which is expressed widely in proximal tubular cells and podocytes. It also can stimulate kidney disease through cytokine-mediated effects and heightened adaptive immune responses [5]. In the current study, we used immunofluorescent staining for the viral spike protein and nucleoprotein, both of which failed to reveal definitive viral protein deposition. In contrast, we observed a significantly higher positive rate of multiple antibodies or antibodies in a non-relevant disease background in the 17 new onset kidney disease patients after vaccination than in pathology-matched kidney disease controls without vaccination (29.41% vs. 8.87%). We also found stronger IgG, IgA or IgM responses to SARS-CoV-2 for immune-complex-mediated glomerular diseases IgAN and LN. We further found that the patients with IgAN and LN after vaccination showed higher levels of pathogenic antibodies compared with matched kidney disease patients enrolled before 2019 (Supplementary Fig. 6). Thus, although the causality between vaccination and these kidney diseases cannot be established, our data support a hypothesis that vaccine-related nephropathy is an immune-driven disease in predisposed individuals. This was consistent with the hypothesis that a neutrophilic immune response to mRNA as a potential trigger plays a role in the *de novo* onset of vasculitis after mRNA-1273 (Moderna) vaccination [24], as immunohistochemical staining for the SARS-CoV-2 spike protein was also negative [25]. The other major disease category in our case series was podocytopathies, including MCD and FSGS, which were identified in five patients. Although our data conclusively demonstrated a lower humoral response in these patients, it is also possible that the cellular immune response might provide protection. Taking CoronaVac (Sinovac) as an example, the protective rate against symptomatic or severe COVID-19 is 83.7%, while its positive cases of humoral responses only account for 50.7%. Actually, it has been reported that inactivated vaccines, such as the influenza vaccine, can trigger many forms of immune-mediated glomerulonephritis, such as MCD [26,27], vasculitis [28], MN [29,30] and Henoch-Schönlein purpura [31]. We await further reports of each type of kidney disease to establish the underlying molecular mechanisms, which could be a greater contribution to the pathogenesis study of immune-complex-mediated kidney disease beyond SARS-CoV-2 vaccines.

In this study, the biopsy-based case series, blood and tissue samples, and the long follow-up time allowed us to understand the pathophysiology and natural history of SARS-CoV-2 vaccine related renal disease. The majority of cases showed a reversible disease course after treatment (Fig. 4). However, it should be noted that data are still limited to prove causality in the cases we present here as well as in other previously published cases. With billions of doses of vaccines being administered worldwide against COVID-19, a coincidence cannot be ruled out. The renal safety of SARS-CoV-2 vaccines has been supported by numbers of previous studies in kidney disease populations. As it was reported that SARS-CoV-2 vaccines were well-tolerated in patients with IgAN, on dialysis or receiving kidney transplantation (Supplementary Table 7). By contrast, hospitalized patients with SARS-CoV-2 infection were reported to show relatively high incident rates of acute kidney injury regardless of medical history of chronic kidney disease, ranging from 1.92% to 80.28%, which was associated with poor prognosis (Supplementary Table 8). In this regard, the Immunonephrology Working Group (IWG) of the European Renal Association-European Dialysis and Transplant Association (ERA-EDTA) stated that `the potential of COVID-19 vaccines to induce immunity protecting from severe COVID-19 should outweigh potential risks in most cases' and `We do recommend vaccination for everyone (except for those with known allergic reactions to any of the vaccine components)'. Several limitations should also be noted. First, this is not a strict population-based study giving an unbiased assessment of the incidence rate of vaccine-related nephropathy. Instead, our case series highlight the need for a stricter monitoring of symptoms following COVID-19 vaccination in participants. Second, this was a hospital-based cohort study, and the time delays for testing for SARS-CoV-2 specific antibodies after vaccination were relatively long. Analogous to natural infection, after three weeks the initial IgM boost response should have subsided in most patients, while the IgG response would be at its peak. Thus, in theory our results might underestimate pathological immune responses. However, the patients did not achieve remission before admission and showed comparable antibody titers with positive controls, whose blood sampling time was around 30 days after vaccination, suggesting that our results could represent the immune response to a certain degree. Third, our testing system only tested humoral (antibody), but not cellular (T cell) immune responses. The cellular part of the adaptive immune system may also play a role in pathogenesis, which is not reflected in our investigation.

In conclusion, our systematic review and biopsy series reveals diverse kidney pathology in *de novo* onset of kidney diseases after vaccination. The kidney diseases after SARS-CoV-2 vaccine show a high consistency across different types of SARS-CoV-2 vaccines. The lack of definitive SARS-CoV-2 proteins in the kidney biopsy samples argues against a direct effect of the vaccine as the major pathomechanism. The serological findings and the findings with regard to the immune response to SARS-CoV-2 highlight the potential for the vaccine to influence innate or adaptive immune responses that in turn trigger the onset of kidney diseases in predisposed individuals. Nephrologists should be aware of this rare but generally treatable potential complication of SARS-CoV-2 vaccinations. Meanwhile, these isolated reports should not lead to vaccination hesitation during this pandemic, because the benefits of vaccination strongly outweigh the potential risks.

## MATERIALS AND METHODS

### Patients and controls

In this clinical-based prospective cohort study, we screened patients at the Renal Division of Peking University First Hospital from 1 January to 10 August 2021 who presented with kidney abnormalities within one month after inactivated SARS-CoV-2 vaccination. Then, we analyzed their baseline information, including sex, age, comorbidities, time of onset, admission delay, diagnostic delay and follow-up data, and compared with the reported cases where it is available. Time of onset was defined as the time at which the patient first noticed symptoms after vaccination. Admission delay was defined as the time from disease onset to hospital admission. Diagnostic delay was defined as the time from disease onset to a confirmed diagnosis of kidney disease. To summarize the prognosis outcome of these patients, we classified them into three groups, ‘remission’, ‘stable’ or ‘dialysis’, which were defined by improvements of renal function or reductions of pathogenic biomarkers (Supplementary Table 5). We collected their blood and urine samples on the day of renal biopsy. Blood samples collected from volunteers who showed no significant adverse effects after they received inactivated SARS-CoV-2 vaccines were used as controls. To exclude any decisive effects of time interval after vaccination, we tried to collect blood samples of these volunteers at the same period after vaccination with the 17 patients. In addition, archived serum samples collected from healthy donors, and serum and urine samples from pathology-matched kidney disease patients enrolled in 2019 (before the emergence of COVID-19) were used as controls.

Kidney biopsies were processed via standard techniques for light microscopy, immunofluorescence and electron microscopy. Immunofluorescent staining was done for IgG, IgA, IgM, complement C3, complement C1q, fibrinogen (FRA), albumin (ALB) and kappa and lambda light chains of immunoglobulins. We also performed immunohistochemical staining for complement C4d and PLA2R.

This study was approved by the institutional review board of Peking University First Hospital (2021-352) and the Committee on Human Subject Research and Ethics of Yunnan University (CHSRE2021020).

### Immunostaining of SARS-CoV-2 spike protein and nucleoprotein

We used formalin-fixed, paraffin-embedded (FFPE) kidney tissue sections from 14 out of the 17 patients with new-onset kidney disease after vaccination and 14 pathology-matched disease controls without vaccination for immunofluorescent staining. According to the manufacturers’ instructions, we selected monoclonal antibodies (mAbs) recognizing the SARS-CoV-2 spike protein (mouse monoclonal IgG1 from clone 1A9; catalog no. GTX632604; GeneTex, Irvine, CA, USA) and nucleoprotein (rabbit monoclonal antibodies from clone 019; catalog no. 40143-R019-B; Sino Biologic, Beijing, People's Republic of China) for staining. Subsequently, we stained the kidney biopsy samples with anti-SARS-CoV-2 spike and anti-SARS-CoV-2 nucleoprotein antibodies at 4°C overnight, and then stained them with fluorescent-labeled secondary antibodies. Mouse lung tissues showing the presence of SARS-CoV-2 were used as positive controls and normal mouse lung tissues were used as negative controls.

### Measurement of SARS-CoV-2-specific antibodies

We collected serum samples via centrifugation of whole blood in test tubes at 1000 × *g* for 15 minutes at room temperature and stored them at −80°C until testing. SARS-CoV-2-specific IgG, IgA and IgM antibodies were measured to profile the humoral immune response to the inactivated SARS-CoV-2 vaccines. We used a magnetic particle chemiluminescence immunoassay (MCLIA, Bioscience Co., Tianjin, China) to measure SARS-CoV-2-specific IgG, IgA and IgM [32,33]. We tested the antibody titer once per serum sample, except for samples that exhibited neutralizing antibody levels higher than a signal-to-cut-off ratio of 30 (the upper boundary of linearity for antibody tests). For these samples, we tested series-diluted samples until the measured values fell within the measurement range.

Antibody titers are presented as the measured chemiluminescence values divided by the cut-off (S/CO). The cut-off value of this test was defined by the receiver operating characteristic curves, and the seroconversion cut-off was defined as 1 S/CO for IgG, IgA and IgM according to the kit manufacturer.

### Measurement of galactose-deficient IgA1

We measured the galactose-deficient IgA1 levels in the vaccine-induced IgAN patients and matched controls using a KM55 ELISA kit (IBL, Japan) [34]. Specifically, we used 1 : 400 diluted serum samples for measurement. The reference range of galactose-deficient IgA1 levels was calculated according to the respective standard curves generated from parallel working standards.

### Statistical analysis

We expressed data as means ± standard deviations (SDs) for normally distributed continuous variables, medians (with IQRs) for non-normally distributed continuous variables and numbers (%) for categorical variables. Then we analyzed the differences across different groups using one-way analysis of variance (ANOVA) for normally distributed continuous variables, a non-parametric test for non-normally distributed continuous variables and Pearson's chi-squared test for the categorical variables. We performed all analyses using GraphPad Prism version 7.0 (GraphPad Software, San Diego, CA, USA) and considered a two-tailed *P* value < 0.05 as statistically significant.

## Supplementary Material

nwac034_Supplemental_File
