# Supplementary material for: Clinicopathological and immunological features of new onset kidney disease: a rare event after SARS-CoV-2 vaccination
Source: Natl Sci Rev. 2023 Apr 14;10(5):nwac034. doi: 10.1093/nsr/nwac034 (PMC10232013; doi:10.1093/nsr/nwac034)
Supplement: nwac034_Supplemental_File [file nwac034_supplemental_file.docx]

**Supplementary Tables and Figures**

**Supplementary table 1. Case reports of kidney diseases after SARS-CoV-2 vaccination in the literature till March 2022.**

| **Number** | **Disease** | **Age** | **Sex** | **Manufacture** | **Dose of disease onset** | **Time between disease onset and vaccination (days)** | **Initial presentation** | **Pathology findings after vaccination** | **Treatment regimen modification** | **Response** | **PMID** |
| --- | --- | --- | --- | --- | --- | --- | --- | --- | --- | --- | --- |
| 1 | de novo MCD | 77 | M | Pfizer-BioNTech | 1 | 7 | NS, AKI | MCD with ATI | pulse MP+CS | PR | 34000278 |
| 2 | de novo MCD | 63 | F | Moderna | 1 | <7 | NS, AKI | MCD with ATI and focal AIN | pulse MP+CS | not reported | 34048824 |
| 3 | de novo MCD | 50 | M | Pfizer-BioNTech | 1 | 4 | NS, AKI | MCD with ATI | CS | CR | 33839200 |
| 4 | de novo MCD | 80's | M | Pfizer-BioNTech | 1 | 7 | NS | MCD | CS | CR | 33992727 |
| 5 | de novo MCD | 61 | F | Pfizer-BioNTech | 1 | 8 | NS, AKI | MCD | CS+dialysis | PR, hemodialysis could be stopped | 34119510 |
| 6 | de novo MCD | 51 | M | Janssen | 1 | 7 | NS, MH | MCD | MP+CS | CR | 34342187 |
| 7 | de novo MCD | 55 | F | Pfizer-BioNTech | 1 | 4 | NS | MCD | CS | CR | 35056345 |
| 8 | de novo MCD | 43 | M | Moderna | 1 | 7 | NS, MH | MCD with IgAN | CS | PR | 34763669 |
| 9 | de novo MCD | 78 | M | Pfizer-BioNTech | 1 | 4 | NS | MCD | CS | PR | 34919048 |
| 10 | de novo MCD | 31 | F | Janssen | 1 | 6 | NS | MCD | CS+RTX | CR | 34919048 |
| 11 | de novo MCD | 80's | F | Pfizer-BioNTech | 1 | 2 | NS, AKI | MCD with AIN | CS | CR | 35246429 |
| 12 | de novo MCD | 31 | F | Pfizer-BioNTech | 2 | 19 | NS | MCD | CS | improved | 34779088 |
| 13 | de novo MCD | 55 | M | AstraZeneca | 2 | 7 | Proteinuria, AKI | MCD with AIN | CS | improved | 34779088 |
| 14 | de novo MCD | 52 | M | Janssen | 1 | 7 | NS | MCD | CS | CR | 35214760 |
| 15 | MCD relapse | 40's | M | Pfizer-BioNTech | 1 | 1 | NS | NA | CS+CsA | CR | 35246429 |
| 16 | MCD relapse | 34 | F | Pfizer-BioNTech | 1 | 10 | SRP, edema | NA | increase of CS dose | CR | 33964312 |
| 17 | MCD relapse | ~ 65 | M | Pfizer-BioNTech | 1 | 8 | NS | NA | CS+CsA | CR | 34023417 |
| 18 | MCD relapse | 30 | M | AstraZeneca | 1 | 2 | SRP | NA | CS | CR | 34119512 |
| 19 | MCD relapse | 40 | F | AstraZeneca | 1 | 1 | Proteinuria, edema | NA | CS | CR | 34119512 |
| 20 | MCD relapse | 22 | M | Pfizer-BioNTech | 1 | 3 | NS | NA | increase of CS dose | CR | 34052236 |
| 21 | MCD relapse | 71 | M | AstraZeneca | 1 | 13 | NS, AKI | MCD+ATI | pulse MP+CS+hemodialysis | CR and hemodialysis was discontinued | 34242687 |
| 22 | de novo FSGS | 20 | F | Pfizer-BioNTech | 1 | 5 | NS | FSGS | CS | PR | 34919048 |
| 23 | de novo IgAN | 30 | M | Moderna | 2 | 1 | GH, SRP | IgAN (M1E0S1T0C0) | RASi | proteinuria reduction | 34278290 |
| 24 | de novo IgAN | 39 | M | Moderna | 2 | <1 | AKI, NS | severe crescentic IgAN | CS+CTX | remission of proteinuria, serum creatinine normalized, but MH persisted | 34087251 |
| 25 | de novo IgAN | 50 | F | Moderna | 2 | 2 | GH, AKI, SRP | IgAN (M1E0S1T1C1) | none | remission of GH in 5 days | 34146600 |
| 26 | de novo IgAN | 39 | F | Moderna | 2 | 2 | MH, SRP | NA | none | spontaneously regressed | 34179593 |
| 27 | de novo IgAN | 19 | M | Moderna | 2 | 2 | GH | IgAN (M1E1S1T0C0) | none | remission of GH in 3 days | 34146600 |
| 28 | de novo IgAN | 41 | F | Pfizer-BioNTech | 2 | 1 | MH, AKI,SRP | IgAN with fibrocelular and fibrous crescents | pulse MP+CS+CTX | not reported | 34033857 |
| 29 | de novo IgAN | 17 | M | Pfizer-BioNTech | 2 | <1 | GH, AKI, SRP | IgAN with cellular glomerular crescents and moderate to severe tubulointerstitial scarring | pulse MP | spontaneously regressed of GH, serum creatinine improved | 34237324 |
| 30 | de novo IgAN | 17 | M | Pfizer-BioNTech | 2 | 2 | GH | IgAN | CS+tonsillectomy | not reported | 34865167 |
| 31 | de novo IgAN | 42 | F | Moderna | 1 | 1 | GH | IgAN | RASi | PR | 35214760 |
| 32 | de novo IgAN | 17 | F | Pfizer-BioNTech | 1 | 4 | Proteinuria, GH | IgAN | none | spontaneous remission | 35118635 |
| 33 | de novo IgAV | 94 | M | Moderna | 2 | 10 | Purpura | NA | CS | disappearance of the purpura, urinalysis and serum creatinine normalized | 34779011 |
| 34 | de novo IgAV | 76 | F | AstraZeneca | 1 | 7 | Purpura, MH | NA | Paracetamol+Deflazacort | CR | 34696186 |
| 35 | de novo IgAV | 50 | M | Pfizer-BioNTech | 1 | 14 | NRP, skin rash | IgAN | CS+ACEI | disappearance of the purpura, renal function improving | 34984055 |
| 36 | de novo IgAV | 47 | M | Moderna | 1 | 19 | purpura, Proteinuria, MH | IgAN | MP+CS | Serum creatinine and proteinuria improved, but MH persisted | 35075622 |
| 37 | IgAN relapse | NA | M | Pfizer-BioNTech | 2 | 5 | AKI, GH | NA | CS | proteinuria and renal function returned to baseline | 34352309 |
| 38 | IgAN relapse | NA | M | Pfizer-BioNTech | 2 | <1 | GH | NA | RASi | spontaneous remission | 34352309 |
| 39 | IgAN relapse | 28 | F | Pfizer-BioNTech | 2 | <1 | GH | IgAN | Not reported | Improved | 34766415 |
| 40 | IgAN relapse | 58 | F | Pfizer-BioNTech | 2 | 1 | GH | NA | Not reported | Improved | 34766415 |
| 41 | IgAN relapse | 52 | F | Pfizer-BioNTech | 2 | <1 | GH, NRP | NA | RASi maintained | remission of GH | 33932458 |
| 42 | IgAN relapse | 38 | F | Moderna | 2 | <1 | GH, SRP | NA | not reported | remission of GH | 33771584 |
| 43 | IgAN relapse | 38 | F | Moderna | 2 | <1 | GH | NA | not reported | remission of GH | 33771584 |
| 44 | IgAN relapse | 22 | M | Moderna | 1 | 2 | GH | NA | none | spontaneously regressed | 34087252 |
| 45 | IgAN relapse | 41 | F | Pfizer-BioNTech | 1 | 2 | GH | NA | none | spontaneously regressed | 34087252 |
| 46 | IgAN relapse | 27 | F | Pfizer-BioNTech | 2 | 2 | GH | NA | none | spontaneously regressed | 34087252 |
| 47 | IgAN relapse | 50 | M | Moderna | 2 | 1 | MH, NRP | Active and chronic IgAN with 13% active crescents | RASi | hematuria and proteinuria returned to baseline; serum creatinine improving but above baseline | 34179593 |
| 48 | IgAN relapse | 13 | M | Pfizer-BioNTech | 2 | <1 | GH, AKI | NA | RASi maintained | spontaneously regressed | 34237324 |
| 49 | de novo GN vasculitis | 77 | M | AstraZeneca | 1 | 28 | AKI | GN vasculitis | MP | serum creatinine normalized within 4 weeks | 34237323 |
| 50 | IgAV relapse | 23 | M | Pfizer-BioNTech | 2 | <1 | Purpura, GH | NA | CS | CR | 34848431 |
| 51 | IgAV relapse | 22 | F | Moderna | 2 | 2 | MH, SRP | NA | none | spontaneously regressed | 34179593 |
| 52 | IgAV relapse | 67 | M | Moderna | 1 | 30 | MH, SRP | NA | CS | hematuria and proteinuria returned to baseline; serum creatinine improving but above baseline | 34179593 |
| 53 | IgAV relapse | 78 | F | Moderna | 1 | 7 | AKI, purpura | NA | MP | disappearance of the purpura, renal function improving | 34250509 |
| 54 | IgAV relapse | 67 | F | Pfizer-BioNTech | 2 | <1 | NS, AKI, purpura | IgAN with necrotizing crescent formation | MP+CTX→CS+azathioprine | renal function improved, but MH and proteinuria persisted | 35275366 |
| 55 | de novo AAV | 81 | M | Moderna | 2 | <1 | AKI, SRP | PR3-ANCA GN | CS+CTX+PE | improved | 34087251 |
| 56 | de novo AAV | 58 | M | Moderna | 2 | 4 | AKI, SRP | anti-PR3 ANCA-associated pauci immune GN | CS+CTX+RTX+PE | CR | 34956751 |
| 57 | de novo AAV | 63 | M | AstraZeneca | 1 | <7 | Hemoptysis, proteinuria, AKI | ANCA-associated pauci-immune GN | CS+CTX | serum creatinine decreased from 257 to 185 μmol/L | 34416184 |
| 58 | de novo AAV | 70 | F | Moderna | 1 | 7 | ARF, GH, proteinuria, hemoptysis | Acute severe renal vasculitis with pauci-immune crescent GN | CS+RTX+PE | hemoptysis resovled, renal function and proteinuria improved, but MH persisted | 34859017 |
| 59 | de novo AAV | 52 | M | Moderna | 2 | 14 | AKI, MH | PR3-ANCA GN, pauci-immune necrotizing and crescentic GN | pulse MP+RTX+CTX | dialysis | 34081948 |
| 60 | de novo renal limited AAV | 78 | F | Pfizer-BioNTech | 1 | <14 | AKI, MH, SRP | Pauci-immune crescentic necrotizing GN | CS+RTX | improved | 34280507 |
| 61 | AAV relapse | 75 | M | AstraZeneca | 1 | 35 | Haemoptysis, MH | Active, pauci‐immune crescentic GN | pulse MP+RTX | dialysis dependent | 34755433 |
| 62 | AAV relapse | 74 | M | AstraZeneca | 1 | 14 | Kidney impairment | Acute pauci‐immune crescentic GN | pulse MP+CTX | renal function improved | 34755433 |
| 63 | de novo anti-GBM-GN | older | F | Moderna | 2 | 14 | AKI, GH, SRP | anti-GBM, coexistent mesangial IgA deposits | CS+CTX+PE | dialysis | 34119511 |
| 64 | de novo anti-GBM-GN | 60 | F | Pfizer-BioNTech | 2 | 1 | MH, NRP, AKI | anti-GBM GN | pulse MP+CS+CTX+PE | not reported | 34033857 |
| 65 | de novo MN | 70 | M | Pfizer-BioNTech | 1 | 7 | NS | PLA2R-negative MN | RASi | no signs of spontaneous remission after 2 months | 34332960 |
| 66 | de novo MN | 76 | M | Cominarty | 1 | 4 | NS, edema | NA (anti-PLA2R antibody 1:800) | RASi+RTX | PR | 34419553 |
| 67 | MN relapse | 66 | F | Sinovac | 1 | 14 | AKI, NRP | PLA2R-associated MN | not reported | not reported | 33992674 |
| 68 | de novo LN | 60 | F | AstraZeneca | 2 | <1 | NRP with multi-organ involved | Class III LN | MP+CTX+CS | improved | 35108572 |
| 69 | de novo LN | 23 | F | AstraZeneca | 1 | 7 | NS with multi-organ involved | Class V LN | MMF+CS | improved | 34560139 |
| 70 | LN relapse | 42 | F | Pfizer-BioNTech | 1 | 7 | NS | Class II and V LN | MMF+CS | remission of ANA titers, but proteinuria persisted | 34352310 |
| 71 | LN relapse | 30 | M | Moderna | 1 | 3 | NRP, AKI, and systemic symptoms | NA | MMF+CS | CR | 34791449 |
| 72 | de novo collapsing GN | 63 | F | AstraZeneca | 1 | 9 | NS | Collapsing GN | CS | CR | 34995650 |
| 73 | de novo collapsing GN | 58 | F | AstraZeneca | 1 | <21 | Edema, acute-on-chronic kidney disease | Collapsing GN | Dialysis | dialysis dependent | 34995650 |
| 74 | de novo TMA | 69 | F | AstraZeneca | 1 | 1 | AKI | TMA | none | spontaneous remission | 35214760 |
| 75 | de novo ATIN | 58 | M | Sinovac | 1 | 9 | AKI, vascular purpura | ATIN | MP+CS | vascular purpura resolved but chronic renal failure remained. | 35074300 |
| 76 | de novo ATIN | 44 | M | Moderna | 1 | 1 | AKI | ATIN | CS | PR | 35214760 |
| 77 | de novo ATIN with  myoglobin  tubular casts | 77 | F | Pfizer-BioNTech | 1 | 1 | AKI | AIN | CS+hemodialysis | PR | 35214760 |
| 78 | IgG4RD relapse | 66 | M | Pfizer-BioNTech | 2 | 14 | AKI | NA | CS+RTX | CR | 34116086 |
| 79 | Acute rejection | 23 | F | Pfizer-BioNTech | 2 | 8 | AKI | Cellular acute rejection | pulse MP+CS+tacrolimus+MMF | serum creatinine decreased from 360 to 230 | 33932459 |

**Abbreviations:** ACEI, angiotension converting enzyme inhibitors; AIN, acute interstitial nephritis; AKI, acute kidney injury; ANCA, anti-neutrophil cytoplasmic antibody; ARF, acute renal failure; ATI, acute tubular injury; CR, complete remission; CS, corticosteroids; CsA, cyclosporine; CTX, cyclophosphamide; GBM, glomerular basement membrane; GH, gross hematuria; GN, glomerulonephritis; MCD, minimal change disease; MH, microscopic hematuria; MMF, Mycophenolate mofetil; MP, methylprednisolone; NA, not available (no biopsy performed); IgAN, IgA nephropathy; IgG4RD, IgG4 related disease; LN, lupus nephritis; NRP, nephrotic range proteinuria; PE, plasma exchange; PLA2R, phospholipase A2 receptor; MN, membranous glomerulopathy; PR, partial response; RASi, renin-angiotensin system inhibitor; RTX, ribuximab; SARS-CoV-2, severe acute respiratory syndrome coronavirus 2; SRP, subnephrotic range proteinuria.

**Supplementary Table 2. Clinical characteristics of the 17 new onset kidney disease patients after inactivated SARS-CoV-2 vaccination in this study.**

| **ID** | **Disease** | **Sex** | **Age** | **Medical history** | **Manufacture**  **(first/second dose)** | **Dose of disease onset** | **Time between disease onset and vaccination (days)** | **Initial manifestations** | **Renal presentations** | | | | | **Complete blood counts** | | | | | **Other laboratory test*** |
| --- | --- | --- | --- | --- | --- | --- | --- | --- | --- | --- | --- | --- | --- | --- | --- | --- | --- | --- | --- |
|  |  |  |  |  |  |  |  |  | **sCr** | **Dialysis** | **Alb** | **24-UTP** | **Urine RBC** | **Hb** | **WBC** | **LY** | **LY%** | **PLT** |  |
| 1 | IgAN | M | 37 | Proteinuria | SinoVac/ SinoVac | 1 | 1 | GN | 101 | N | 44.1 | 1.56 | 3~5 | 166 | 9.05 | 2.71 | 29.9 | 336 | - |
| 2 | IgAN | F | 27 | N | Sinopharm/ Sinopharm | 2 | 1 | E, GN, AKI | 133 | N | 27.8 | 2.22 | 10~20 | 144 | 7.45 | 2.53 | 34 | 333 | IgA 5.76↑  IgG 5.72↓ |
| 3 | IgAN+ATIN | M | 60 | Hypertension, Fatty liver, Gout | SinoVac / Sinopharm | 2 | 1 | E, GN, AKI | 258.6 | N | 31.5 | 9.38 | 5~8 | 136 | 5.5 | 1.0 | 18.5 | 211 | IgA 4.10↑ |
| 4 | MN (I) | M | 42 | Smoking | Sinopharm / SinoVac | 1 | 4 | NRP | 90 | N | 37.2 | 11.27 | 0~2 | 164 | 6.74 | 1.84 | 27.3 | 170 | IgG 5.42↓  IgM 0.4↓ |
| 5 | MN (I~II) | F | 33 | Proteinuria, Hypertension | SinoVac | 1 | 17 | SRP | 58.4 | N | 33.1 | 2.89 | 0 | 141 | 4.4 | 1.07 | 24.4 | 247 | ANA 1:3200  Anti-SS-A 113↑ |
| 6 | SLE, LN-IV | M | 33 | N | SinoVac/ SinoVac | 1 | 1 | Butterfly rash, proximal interphalangeal joint swelling, NS, MH | 118.37 | N | 28.1 | 12.37 | 15~20 | 134 | 4.11 | 1.13 | 27.5 | 136 | ANA 1:10000  Anti-dsDNA 791↑  Anti-HIS 42↑  Anti-ANuA 30↑  Anti-PCNA 29↑  C3 0.279↓  C4 0.039↓ |
| 7 | SLE, LN-V | M | 45 | Pruritus | SinoVac / SinoVac | 2 | 2 | Alopecia, photosensitivity, joint pain, GN | 81.3 | N | 31.1 | 5.04 | 20~25 | 143 | 3.20 | 0.70 | 21.9 | 151 | ANA 1:1000  Anti-SS-A 155↑  Anti-rRNP 149↑ |
| 8 | MCD | F | 39 | N | SinoVac | 1 | 4 | NS | 64.3 | N | 18.6 | 6.12 | 0~2 | 135 | 4.8 | 0.9 | 19.8 | 225 | ANA 1:100  Anti-Jo-1 39↑  IgA 4.47↑  IgM 2.83↑ |
| 9 | MCD+IgAN | F | 39 | Asthma, Rhinitis, Gall-stone | SinoVac / SinoVac | 2 | 14 | Purpura, E, NS | 69 | N | 27.7 | 4.32 | 0~2 | 161 | 9.46 | 3.47 | 36.6 | 403 | C4 0.498↑ |
| 10 | MCD | M | 30 | Smoking | SinoVac / SinoVac | 2 | 15 | NRP | 64.28 | N | 30.4 | 7.8 | 0 | 164 | 8.86 | 3.17 | 35.7 | 291 | C4 0.418↑ |
| 11 | FSGS(NOS)+ATI+IgAN | F | 26 | Obesity, Smoking | Sinopharm / Sinopharm | 1 | 1 | Diarrhea, E, NS, AKI | 164.9 | N | 17.4 | 26.12 | 15~25 | 132 | 5.75 | 2.12 | 36.9 | 412 | IgG 1.87↓  C4 0.374↑ |
| 12 | FSGS(Tip) | F | 79 | Hypertension, Gastroesophageal reflux disease | Sinopharm / Sinopharm | 2 | 20 | E, NS | 68 | N | 29.1 | 4.34 | 3~5 | 129 | 5.5 | 1.13 | 20.5 | 294 | - |
| 13 | AAV | F | 65 | Hypertension | SinoVac / SinoVac | 2 | 3 | RPGN | 336.9 | N | 35.3 | 0.56 | >100 | 105 | 13.98 | 1.30 | 9.3 | 420 | MPO-ANCA 494.50↑ |
| 14 | AAV | M | 85 | Arthrodynia | Sinopharm / Sinopharm | 2 | 14 | Hemoptysis, RPGN | 438.87 | Y | 32.2 | 1.69 | 200~250 | 99 | 7.0 | 0.5 | 7.3 | 128 | MPO-ANCA >200↑ |
| 15 | anti-GBM GN+AIN | M | 39 | N | SinoVac / SinoVac | 2 | 1 | Fever, diarrhea, RPGN | 404.4 | N | 32 | 1.3 | 200~300 | 110 | 22.9 | 2.6 | 11.3 | 390 | Anti-GBM 123↑  PLA2R 37↑ |
| 16 | ATI+AIN | F | 68 | Proteinuria, Hypertension, Diabetes, Cataract | Sinopharm | 1 | 3 | Fever, purpura, AKI | 224 | N | 36.8 | - | 0 | 111 | 10.58 | 0.87 | 8.2 | 517 | IgA 5.79↑ |
| 17 | TMA | F | 50 | Psoriasis | SinoVac / Sinopharm | 2 | 7 | Proteinuria, high blood pressure, AKI | 546 | Y | 32.5 | 0.11 | 40 | 90 | 9.27 | 0.62 | 6.7 | 104 | ANA 1:100  C3 0.552↓ |

**Note:** Other laboratory test includes IgA (g/L, range 0.69-3.82), IgG (g/L, range 7.23-16.85), IgM (g/L, range 0.63-2.77), C3 (g/L, range 0.6-1.5), C4 (g/L, range 0.12-0.36), ANCA (negative), PR3 (<20), MPO (<20), anti-GBM (<20), HBsAg (<0.05 IU/mL), HBeAg (<1.0 S/CO), anti-HCV (<1.0 S/CO), anti-HIV (<1.0 S/CO), TP-Ab (<1.0 S/CO), ANA (1:<100), anti-dsDNA (<100 IU/mL), anti-nRNP (<25), anti-Sm (<25), anti-SS-A (<25), anti-SS-B (<25), anti-Scl-70 (<25), anti-Jo-1 (<25), anti-rRNP, anti-AMA-M2 (<25), anti-HIS (<25), anti-ANuA (<25), anti-CENP B (<25), anti-PCNA (<25), and PLA2R (<20 RU/mL).

**Abbreviations:** AAV, anti-neutrophil cytoplasmic antibody (ANCA)-associated vasculitis; AIN, acute interstitial nephritis; AKI, acute kidney injury; Alb, albumin (g/l); ATI, acute tubular injury; ATIN, acute tubular-interstitial nephropathy; E, edema; F, female; FSGS, focal segmental glomerulosclerosis; GBM, glomerular basement membrane; GN, glomerulonephritis; Hb, hemoglobin (g/l); ID, identification number; IgAN, IgA nephropathy; LN, lupus nephritis; LY, lymphocyte (*10^9/L); M, male; MCD, minimal change disease; MH, microscopic hematuria; MN, membranous nephropathy; N, No; NRP, nephrotic range proteinuria; NS, nephrotic syndrome; PLT, platelet (*10^12/L); RBC, red blood cell; RPGN, rapidly progressive glomerulonephritis; SARS-CoV-2, severe acute respiratory syndrome coronavirus 2; sCr, serum creatinine (μmol/l); SRP, sub-nephrotic range proteinuria; TMA, thrombotic microangiopathy; UTP, urinary total protein (g/d); WBC, white blood cell (*10^9/L); Y, Yes.

**Supplementary Table 3. Serological data of the 282 patients with kidney diseases enrolled in 2019.**

| **Diagnosis** | **ANA** | **dsDNA** | **ENA** | | | | | | | |  |  |  |
| --- | --- | --- | --- | --- | --- | --- | --- | --- | --- | --- | --- | --- | --- |
|  |  |  | **nRNP** | **Sm** | **SS-A/B** | **Scl** | **rRNP** | **AMA-M2** | **HIS** | **CENP B** | **MPO-ANCA** | **PR3-ANCA** | **PLA2R** |
| **IgAN (10/115)** | | | | | | | | | | | | | |
| **1** | 1:1000 | - | - | - | - | - | - | - | - | 120 | - | - | - |
| **2** | 1:100 | 101 | - | - | - | - | - | - | - | - | - | - | - |
| **3** | 1:100 | - | 35 | - | - | - | - | - | - | - | - | - | - |
| **4** | 1:1000 | - | - | - | - | - | - | - | - | - | - | 55 | - |
| **5** | 1:3200 | - | - | - | - | - | - | - | - | 201 | - | - | - |
| **6** | - | - | - | - | - | - | - | 62 | - | - | - | - | - |
| **7** | 1:1000 | - | - | - | - | - | - | - | - | - | - | - | - |
| **8** | - | - | - | - | 36 | - | - | - | - | - | - | 71 | - |
| **9** | 1:100 | - | - | - | - | 45 | - | - | - | - | - | - | - |
| **10** | 1:100 | - | - | - | - | - | - | - | 35 | - | - | - | - |
| **MN (3/83)** | | | | | | | | | | | | | |
| **11** | 1:100 | - | - | - | 80 | - | - | - | - | - | - | - | - |
| **12** | 1:320 | - | - | - | - | - | - | - | - | + | - | - | 451 |
| **13** | - | - | - | - | - | - | - | - | - | - | - | 31 | 871 |
| **LN (8/14)** | | | | | | | | | | | | | |
| **14** | 1:10000 | 185 | - | - | 87 | - | 102 | - | - | - | - | - | - |
| **15** | 1:32000 | 139 | - | - | 107 | - | - | - | - | - | - | 62 | - |
| **16** | 1:10000 | 169 | - | - | 129 | - | 71 | - | - | - | - | - | - |
| **17** | 1:1000 | 120 | 29 | - | 111 | - | - | - | - | - | - | - | - |
| **18** | 1:3200 | 128 | - | - | 45 | - | - | - | - | - | - | - | - |
| **19** | 1:3200 | - | - | 27 | 143/118 | - | 49 | - | - | - | - | - | - |
| **20** | 1:10000 | - | 114 | - | 97 | - | - | - | - | - | - | - | - |
| **21** | 1:3200 | - | 125 | 101 | - | - | - | - | - | - | 173 | - | - |
| **MCD (0/14)** | | | | | | | | | | | | | |
| **FSGS (2/24)** | | | | | | | | | | | | | |
| **22** | - | - | - | - | - | - | - | - | - | - | - | - | 92 |
| **23** | - | - | - | - | - | - | - | - | - | - | - | 61 | - |
| **AAV (1/12)** | | | | | | | | | | | | | |
| **24** | 1:100 | - | - | - | - | - | - | 47 | - | - | 85 | - | - |
| **anti-GBM GN (0/5)** | | | | | | | | | | | | | |
| **ATIN (0/11)** | | | | | | | | | | | | | |
| **TMA (1/4)** | | | | | | | | | | | | | |
| **25** | 1:1000 | - | - | - | - | - | - | - | - | - | - | - | - |

**Note:** The 282 patients with the same pathology with Vac-patients (patients presented kidney abnormalities after inactivated SARS-CoV-2 vaccination within one month) were enrolled from January 1st 2019 to April 30th 2019.

**Abbreviations:** AAV, anti-neutrophil cytoplasmic antibody (ANCA)-associated vasculitis; ATIN, acute tubular-interstitial nephropathy; FSGS, focal segmental glomerulosclerosis; GBM, glomerular basement membrane; GN, glomerulonephritis; IgAN, IgA nephropathy; LN, lupus nephritis; MCD, minimal change disease; MN, membranous nephropathy; TMA, thrombotic microangiopathy.

**Supplementary Table 4. Pathologic findings in the 17 patients with kidney diseases after inactivated SARS-CoV-2 vaccination in this study.**

| **ID** | **Diagnosis** | **Time of biopsy** | **LM** | | | | | | | **IHC** | | **IF** | | | | | | | | | **EM** | |
| --- | --- | --- | --- | --- | --- | --- | --- | --- | --- | --- | --- | --- | --- | --- | --- | --- | --- | --- | --- | --- | --- | --- |
|  |  |  | **No.Glom** | **No.GS** | **Hyper-cellularity** | **No.C** | **II** | **IFTA** | **VS** | **C4d** | **PLA2R** | **IgG** | **IgA** | **IgM** | **C3** | **C1q** | **FRA** | **Alb** | **κ** | **λ** | **Dense deposits** | **FPE** |
| 1 | IgAN | 154 | 9 | 1 | Mes | 3 | N | Y | N | 2~3+ | - | ± | 3+ | 1+ | 3+ | - | 1+ | - | 3+ | 3+ | Y | Y |
| 2 | IgAN | 14 | 11 | 7 | Mes | 2 | N | Focal | N | 2+ | - | - | 4+ | 1+ | 3+ | ± | - | - | 3+ | 4+ | Y | Y |
| 3 | IgAN  ATIN | 47 | 16 | 2 | Mes+Endo | 10 | Y | Focal | Y | 2+ | - | - | 3+ | 1+ | 3+ | - | - | - | 2+ | 2+ | Y | Y |
| 4 | MN  (I) | 124 | 40 | 0 | Mes | 0 | N | Focal | Y | 3+ | 3+ | 3+ | - | - | 2+ | - | - | - | 3+ | 3+ | Y | Y |
| 5 | MN  (I~II) | 132 | 8 | 1 | Mes | 0 | N | N | N | 3+ | 3+ | 3+ | 1+ | - | 1+ | - | - | - | 3+ | 3+ | Y | Y |
| 6 | LN-IV | 62 | 28 | 1 | Mes+Endo | 9 | N | Focal | N | 4+ | - | 3~4+ | 2~3+ | 2+ | 3+ | 2+ | - | ± | 3+ | 3+ | Y | Y |
| 7 | LN-V | 18 | 42 | 0 | Mes | 0 | N | N | N | 3+ | - | 3+ | 1+ | - | 3+ | 2+ | - | - | 3+ | 3+ | Y | Y |
| 8 | MCD | 25 | 22 | 0 | Mes | 0 | N | N | Y | 2+ | - | - | - | 1+ | - | - | - | - | - | - | N | Y |
| 9 | MCD  IgAN | 58 | 36 | 0 | Mes | 0 | N | N | Y | 2+ | - | - | 2+ | 1+ | 1+ | - | - | - | 2+ | 2+ | Y | Y |
| 10 | MCD | 59 | 26 | 2 | N | 0 | N | Focal | N | 1+ | - | - | - | ± | - | - | - | - | - | - | Y | Y |
| 11 | FSGS  (NOS)  ATI  IgAN | 37 | 21 | 3 | Mes | 0 | N | Focal | N | 2+ | - | - | 2+ | - | 2+ | - | - | - | 1~2+ | 1+ | Y | Y |
| 12 | FSGS  (Tip) | 15 | 16 | 2 | Mes | 0 | N | Focal | Y | 2+ | - | - | - | - | - | 1+ | - | - | - | - | N | Y |
| 13 | AAV | 39 | 21 | 4 | Endo | 13 | Y | Y | N | - | - | ± | - | - | 1+ | - | 2+ | - | - | - | N | Y |
| 15 | Anti-GBM  AIN | 21 | 9 | 0 | N | 6 | Y | Diffuse | N | NA | NA | 2+ | - | - | - | - | - | NA | NA | NA | NA | NA |
| 16 | ATI  AIN | 33 | 15 | 4 | Mes | 0 | Y | Focal | Y | - | - | - | - | - | - | - | - | - | - | - | N | N |
| 17 | TMA | 40 | 17 | 0 | N | 0 | N | Focal | N | NA | - | - | 1+ | - | - | - | - | NA | - | - | NA | NA |

**Note:** Time of biopsy indicates the time from disease onset to kidney biopsy.

**Abbreviations:** AAV, anti-neutrophil cytoplasmic antibody (ANCA)-vasculitis; Alb, albumin; AIN, acute interstitial nephritis; ATI, acute kidney injury; ATIN, acute tubulointerstitial renal disease; C, crescents; EM, electron microscopy; Endo, endocapillary hypercellularity; FPE, foot process effacement; FSGS, focal segmental glomerulosclerosis; Glom, glomeruli; GS, globular sclerosis; ID, identification number; IF, immune-fluorescent (stain); IFTA, tubular atrophy and interstitial fibrosis; IgAN, IgA nephropathy; IHC, immunohistochemistry; II, interstitial inflammation; LM, light microscopy; LN, lupus nephritis; MCD, minimal change disease; Mes, mesangial hypercellularity; MN, membranous nephropathy; N, No; NA, not available; No, number; SARS-CoV-2, severe acute respiratory syndrome coronavirus 2; TMA, thrombotic microangiopathy; VS, vascular sclerosis; Y, Yes.

**Supplementary Table 5. Treatment and follow-up information of the 17 new onset kidney disease patients after inactivated SARS-CoV-2 vaccination in this study.**

| **ID** | **Disease** | **Therapy** | **Duration** | **sCr** | **Alb** | **24-UTP** | **Urine RBC** | **Autoantibody** | **Outcomes** | **Outcome classification** |
| --- | --- | --- | --- | --- | --- | --- | --- | --- | --- | --- |
| 1 | IgAN | ARB | 165 | 94 | 40.4 | 1.58 | 40~60 | - | Renal function improving | Remission |
| 2 | IgAN | Antibiotics | 109 | 124 | 30.9 | 4.03 | 60~80 | - | Renal function improving | Remission |
| 3 | IgAN+ATIN | Pulse Methylprednisone → Corticosteroids + Cyclophosphamide | 73 | 241 | 33.2 | - | - | - | Renal function improving | Remission |
| 4 | MN (I) | Corticosteroids +Ciclosporin | 129 | 97.45 | 28.4 | - | - | - | Renal function remained stable | Stable |
| 5 | MN (I~II) | ARB | 152 | 56 | 34.8 | 1.66 | 0 | - | Proteinuria decreased from 2.89 to 1.66 | Remission |
| 6 | SLE, LN-IV | Pulse methylprednisone → Methylprednisone / Corticosteroids + Cyclophosphamide + Hydroxychloroquine | 90 | 116.76 | 18.9 | 10.96 | 500~600 | Anti-dsDNA <100  Anti-HIS 38↑  Anti-ANuA 5  Anti-PCNA 73↑  C3 0.457↓  C4 0.131 | Renal function remained stable,  Proteinuria decreased,  Autoantibodies decreased,  C3 and C4 improved | Remission |
| 7 | SLE, LN-V | Corticosteroids +Ciclosporin+ hydroxychloroquine | 169 | 102.85 | 34.7 | 2.42 | 0-2 | - | Proteinuria decreased from 5.04 to 2.42 | Remission |
| 8 | MCD | Corticosteroids | 151 | 69 | 40.8 | 0.09 | 0 | - | Proteinuria decreased to <0.5 | Remission |
| 9 | MCD+IgAN | Corticosteroids | 80 | 65 | 37.3 | 0.14 | 15~20 | - | Proteinuria decreased to <0.5 | Remission |
| 10 | MCD | Corticosteroids | 108 | 81 | 41.1 | 0.05 | 0 | - | Proteinuria decreased to <0.5 | Remission |
| 11 | FSGS (NOS)+ATI+IgAN | Corticosteroids | 70 | 70 | 24.6 | - | - | - | Serum creatinine normalized | Remission |
| 12 | FSGS (Tip) | Tacrolimus, antibiotics | 21 | 67 | 25.5 | 4.5 | - | - | Renal function remained stable | Stable |
| 13 | AAV | Methylprednisone + Cyclophosphamide → Corticosteroids | 60 | 219 | 36.8 | - | 10~15 | MPO-ANCA 135↑ | sCr decreased from 336.9 to 219 MPO-ANCA decreased from 494 to 135 | Remission |
| 14 | AAV | Plasma exchange + Dialysis + Rituximab | 60 | 457.39 | 38.7 | - | - | MPO-ANCA 130↑ | Dialysis dependent,  Disappearance of hemoptysis,  MPO-ANCA decreased from>200 to 130 | Dialysis |
| 15 | Anti-GBM GN+AIN | Plasma exchange+ Corticosteroids + Cyclophosphamide | 70 | 222 | 40.3 | 0.7 | 60~70 | Anti-GBM <20  PLA2R <20 | sCr decreased from 404.4 to 222  Anti-GBM and PLA2R turned to be negative | Remission |
| 16 | ATI+AIN | Corticosteroids | 86 | 118 | 41.2 | - | 0 | - | sCr decreased from 224 to 118 | Remission |
| 17 | TMA | Plasma exchange + Dialysis + ACEI+ MMF | 62 | 641.48 | 26.2 | - | - | - | Dialysis dependent | Dialysis |

**Note:** Duration indicates the time from disease onset to the latest visit (days).

**Abbreviations:** AAV, anti-neutrophil cytoplasmic antibody (ANCA)-associated vasculitis; ACEI, angiotensin converting enzyme inhibitor; AIN, acute interstitial nephritis; Alb, albumin (g/l); ARB, angiotensin receptor blockers; ATI, acute tubular injury; ATIN, acute tubular-interstitial nephropathy; CR, complete remission; FSGS, focal segmental glomerulosclerosis; GBM, glomerular basement membrane; GN, glomerulonephritis; ID, identification number; IgAN, IgA nephropathy; LN, lupus nephritis; MCD, minimal change disease; MMF, mycophenolate mofetil; MN, membranous nephropathy; PLA2R, M-type phospholipase A2 receptor; RBC, red blood cell; SARS-CoV-2, severe acute respiratory syndrome coronavirus 2; sCr, serum creatinine (μmol/l); TMA, thrombotic microangiopathy; UTP, urinary total protein (g/d).

**Supplementary Table 6. The potential disease spectrum of kidney diseases after infecting with SARS-CoV-2 or receiving SARS-CoV-2 vaccines.**

| **Kidney diseases** | **Patients infecting with**  **SARS-CoV-2** | **Patients receiving**  **non-inactivated SARS-CoV-2 vaccines** | **Patients receiving**  **inactivated SARS-CoV-2 vaccines** |
| --- | --- | --- | --- |
| IgAN/IgAV | √ | √ | √ |
| MN | √ | √ | √ |
| LN | √ | √ | √ |
| MCD | √ | √ | √ |
| FSGS | √ | √ | √ |
| Vasculitis | √ | √ | √ |
| Anti-GBM GN | √ | √ | √ |
| ATIN | √ | √ | √ |
| TMA | - | √ | √ |
| IgG4RD | - | √ | - |

**Abbreviations:** ATIN, acute tubular-interstitial nephropathy; FSGS, focal segmental glomerular sclerosis; GBM, glomerular basement membrane; GN, glomerulonephritis; IgAN, IgA nephropathy; IgAV, IgA vasculitis; IgG4RD, IgG4 related disease; LN, lupus nephritis; MCD, minimal change disease; MN, membranous glomerulopathy; SARS-CoV-2, severe acute respiratory syndrome coronavirus 2; TMA, thrombotic microangiopathy.

**Supplementary Table 7. Safety evaluation of SARS-CoV-2 vaccines in patients with kidney diseases in published studies.**

| **Number** | **Number of cases** | **Manufacture** | **Dose** | **Local reactogenicity** | **Systemic reactogenicity** | **Serious adverse events** | **Others** | **Conclusion** | **PMID** |
| --- | --- | --- | --- | --- | --- | --- | --- | --- | --- |
| 1 | dialysis patients (169 on hemodialysis and 21 with peritoneal dialysis) | Pfizer-BioNTech | 1 dose/2dose | Comparable with controls. | Comparable with controls. | No serious adverse events related to vaccination | - | Well-tolerated | 34357013 |
| 2 | 1304 dialysis patients | Pfizer-BioNTech/  Moderna | 1 dose | Pain at the injection site appeared to be less frequent than controls. | Joint pain and chill appeared to be less frequent than controls. | Hospitalization due to vaccination (5/0.4%) | - | Well-tolerated | 34318288 |
| 3 | 1304 dialysis patients | Pfizer-BioNTech/  Moderna | 2 dose | Pain at the injection site appeared to be less frequent than controls. | Joint pain and fever appeared to be less frequent than controls. | Hospitalization due to vaccination (5/0.4%) | - | Well-tolerated |  |
| 4 | 376 kidney transplant recipients | Pfizer-BioNTech/  Moderna | 1 dose | Comparable with controls. | Comparable with controls. | Hospitalization due to vaccination (3/0.8%) | - | Well-tolerated |  |
| 5 | 376 kidney transplant recipients | Pfizer-BioNTech/  Moderna | 2 dose | Comparable with controls. | Joint pain, fever and chill appeared to be less frequent compared to controls. | Hospitalization due to vaccination (6/1.6%) | - | Well-tolerated |  |
| 6 | 83 kidney transplant recipients | AstraZeneca | 1 dose | Well-tolerated | Well-tolerated | No serious adverse events related to vaccination | - | Well-tolerated | 35090091 |
| 7 | 83 kidney transplant recipients | AstraZeneca | 2 dose | Well-tolerated | Well-tolerated | No serious adverse events related to vaccination | - | Well-tolerated |  |
| 8 | 197 kidney transplant recipients | 99 with Pfizer-BioNTech/  Moderna;  98 with Janssen | 3 dose | Only 1% to 2% of patients reported severe fatigue, fever, myalgia, or pain. | | No serious adverse events related to vaccination | - | Well-tolerated | 34928302 |
| 9 | 30 kidney transplant recipients | Pfizer-BioNTech/  Moderna | 2 dose | - | - | No serious adverse events related to vaccination | - | Well-tolerated | 34522992 |
| 10 | 130 kidney transplant recipients | Pfizer-BioNTech | 3 dose | - | - | No serious adverse events related to vaccination | - | Well-tolerated | 35346486 |
| 11 | 443 patients with IgA nephropathy or IgA vasculitis | Sinovac/  BBIBP-CorV | at least 1 dose | - | - | No serious adverse events related to vaccination | eGFR showed temporary decline in a few patients | Well-tolerated | 35399860 |
| 12 | 50 patients on hemodialysis | Pfizer-BioNTech | 1 dose | 38% of patients reported mild pain at injection site. | Systemic reactogenicity occurred less often, with diarrhea (4% mild and 4% moderate) and fatigue (8% mild) being the most frequent ones. | No serious adverse events related to vaccination | - | Well-tolerated | 34220867 |
| 13 | 48 patients on hemodialysis | Pfizer-BioNTech | 2 dose | Injection site pain with 29.2% for mild, 2.1% for moderate, and 2.1% for severe. | Systemic reactogenicity occurred less often, with chill (4.2% mild), headache (4.2% mild), fatigue (4.2% mild), joint pain (2.1% moderate and 2.1% severe), and muscle pain (2.1% severe) being the most frequent ones. | No serious adverse events related to vaccination | - | Well-tolerated |  |
| 14 | 28 patients with peritoneal dialysis | Pfizer-BioNTech/  Moderna/  AstraZeneca | 1 dose | Pain at the injection site appeared to be less frequent than controls. | Headache, chills, fever, fatigue, muscle pain and joint pain appeared to be less frequent than controls. | No serious adverse events related to vaccination | - | Well-tolerated | 34854131 |
| 15 | 23 patients with peritoneal dialysis | Pfizer-BioNTech/  Moderna/  AstraZeneca | 2 dose | Pain at the injection site appeared to be less frequent than controls. | Headache, fatigue and joint pain appeared to be less frequent than controls. | No serious adverse events related to vaccination | - | Well-tolerated |  |

**Note:** Local reactogenicity includes redness, swelling, and pain at the injection site; Systemic reactogenicity includes fever, fatigue, headache, chills, vomiting, diarrhea, muscle pain, and joint pain; Serious adverse events are defined as any untoward medical occurrence that resulted in death, life-threatening, requiring inpatient hospitalization or prolongation of existing hospitalization, or resulted in persistent disability/incapacity.

**Supplementary Table 8. The prevalence of kidney disease in patients infected with SARS-CoV-2 in published studies.**

| **Number** | **Location** | **Population** | **Total number of patients (n)** | **Mortality**  **(n, % of total patients)** | **Incidence of AKI** | | |  | **Baseline CKD** | | |  | **Renal replacement therapy used**  **(n, % of total patients)** | **PMID** |
| --- | --- | --- | --- | --- | --- | --- | --- | --- | --- | --- | --- | --- | --- | --- |
|  |  |  |  |  | **Total (n, % of total patients)** | **ICU or severe disease**  **(n, % of AKI patients)** | **Death**  **(n, % of AKI patients)** |  | **Total**  **(n, % of total patients)** | **ICU or severe disease**  **(n, % of CKD patients)** | **Death**  **(n, % of CKD patients)** |  |  |  |
| 1 | China | Hospitalized | 21 | 4 (19.05) | 2 (9.52) | 2 (100) | - |  | 0 | - | - |  | - | 32217835 |
| 2 | China | Hospitalized | 333 | 29 (8.71) | 22 (6.61) | - | 19 (86.36) |  | 0 | - | - |  | - | 32345702 |
| 3 | China | Hospitalized | 104 | 1 (0.96) | 2 (1.92) | - | - |  | 0 | - | - |  | - | 32369217 |
| 4 | Korea | Hospitalized+ICU | 98 | 5 (5.1) | 9 (9.18) | 8 (88.89) | - |  | 0 | - | - |  | 3 (3.06) | 32390367 |
| 5 | USA | Hospitalized | 5449 | 888 (16.3) | 1993 (36.58) | 1060 (53.19) | 694 (65.47) |  | 0 | - | - |  | 285 (5.23) | 32416116 |
| 6 | UK | Hospitalized | 450 | 70 (15.56) | 85 (18.89) | - | 54 (63.53) |  | 0 | - | - |  | - | 32586323 |
| 7 | Italy | ICU | 99 | 61 (61.62) | 72 (72.73) | 72 (100) | 28 (38.89) |  | 0 | - | - |  | 17 (17.17) | 32659757 |
| 8 | Iran | Hospitalized non-kidney disease | 451 | 105 (23.3) | 156 (34.7) | - | - |  | 0 | - | - |  | - | 34278999 |
|  | Iran | Hospitalized kidney disease | 208 | 73 (35) | 141 (68) | - | - |  | 87 (41.83) | - | - |  | - | 34278999 |
| 9 | China | Hospitalized+ICU | 41 | 6 (14.63) | 3 (7.32) | 3 (100) | - |  | - | - | - |  | 3 (7.32) | 31986264 |
| 10 | China | Hospitalized+ICU | 138 | 6 (4.35) | 5 (3.62) | 2 (40) | - |  | 4 (2.9) | 2 (50) | - |  | 2 (1.45) | 32031570 |
| 11 | China | ICU | 52 | 32 (61.54) | 15 (28.85) | 15 (100) | 12 (80) |  | - | - | - |  | 9 (17.31) | 32105632 |
| 12 | China | Laboratory-confirmed COVID-19 | 1099 | 15 (1.36) | 6 (0.55) | 5 (83.33) | - |  | 8 (0.73) | 3 (37.5) | - |  | 9 (0.82) | 32109013 |
| 13 | China | Hospitalized+ICU | 191 | 54 (28.27) | 28 (14.66) | 28 (100) | 27 (96.43) |  | 2 (1.05) | 2 (100) | 2 (100) |  | 10 (5.24) | 32171076 |
| 14 | USA | ICU | 21 | 11 (52.38) | 4 (19.05) | 4 (100) | - |  | 10 (47.62) | 10 (100) | - |  | - | 32191259 |
| 15 | China | Hospitalized | 416 | 57 (13.7) | 8 (1.92) | - | - |  | 14 (3.37) | - | - |  | 2 (0.48) | 32211816 |
| 16 | China | Deceased+discharged | 274 | 113 (41.24) | 29 (10.58) | - | 28 (96.55) |  | 4 (1.46) | - | - |  | - | 32217556 |
| 17 | China | Hospitalized | 339 | 65 (19.17) | 27 (7.96) | - | 17 (62.96) |  | 13 (3.83) | - | 4 (30.77) |  | - | 32240670 |
| 18 | China | Hospitalized | 701 | 113 (16.12) | 36 (5.14) | - | - |  | 14 (2) | - | - |  | - | 32247631 |
| 19 | USA | Hospitalized | 5700 | 553 (9.7) | 1370 (24.04) | - | - |  | 454 (7.96) | - | - |  | 225 (3.95) | 32320003 |
| 20 | China | Deceased+discharged | 107 | 19 (17.76) | 14 (13.08) | - | 14 (100) |  | 3 (2.8) | - | 1 (33.33) |  | - | 32354360 |
| 21 | China | ICU | 34 | 0 | 7 (20.59) | 7 (100) | - |  | 2 (5.89) | 2 (100) | - |  | 5 (14.71) | 32425003 |
| 22 | France | ICU | 71 | 4 (5.63) | 57 (80.28) | 57 (100) | 4 (7.02) |  | 4 (5.63) | 4 (100) |  |  | 10 (14.08) | 32695326 |
| 23 | USA | Hospitalized | 3993 | 1085 (27.17) | 1835 (45.96) | 745 (40.6) | 918 (50.03) |  | 420 (10.52) | - | - |  | - | 32883700 |
| 24 | Swiss | Hospitalized | 145 | 14 (9.66) | 35 (24.14) | - | - |  | 15 (10.34) | - | - |  | - | 33186355 |
| 25 | China | Hospitalized | 287 | 19 (6.62) | 55 (19.16) | 34 (61.82) | 12 (21.82) |  | 5 (1.74) | - | - |  | - | 33624587 |
| 26 | USA | Hospitalized | 9657 | 2418 (25.04) | 3854 (39.91) | 2042 (52.98) | 1491 (38.69) |  | 492 (5.09) | - | - |  | - | 32961245 |
| 27 | Spain | Hospitalized | 1603 | (12.3) | 235 (14.66) | - | - |  | (8.5) | - | - |  | - | 32871592 |
| 28 | Italy | Hospitalized | 307 | 55 (17.92) | 69 (22.48) | - | 39 (56.52) |  | 51 (16.61) | - | - |  | 5 (1.63) | 34196877 |
| 29 | UK | Hospitalized | 1161 | 419 (36.09) | 304 (26.18) | 64 (21.1) | 184 (60.5) |  | 224 (19.29) | - | - |  | 23 (1.98) | 33125416 |
| 30 | Germany | ICU | 37 | 9 (24.32) | 28 (75.68) | - | 9 (32.14) |  | 22 (59.46) | - | - |  | 22 (59.46) | 34015009 |
| 31 | UK | Hospitalized | 1855 | 574 (30.94) | 455 (24.5) | 166 (36.48) | 265 (58.24) |  | 248 (13.37) | - | - |  | 74 (3.99) | 34751235 |
| 32 | USA | Hospitalized | 8121 | 1579 (19.44) | 2110 (25.98) | 1038 (49.19) | 825 (39.1) |  | 1176 (14.48) | - | - |  | 315 (3.88) | 34107743 |
| 33 | China | Hospitalized | 1708 | 76 (4.45) | 115 (6.73) | 51 (44.35) | 37 (32.17) |  | 43 (2.52) | - | - |  | - | 34312430 |
| 34 | Turky | Hospitalized | 621 | 86 (13.85) | 202 (32.53) | 85 (42.08) | 68 (33.66) |  | 304 (48.95) | - | - |  | - | 33611868 |
| 35 | USA | Hospitalized+ICU | 575 | 160 (27.83) | 161 (28) | 105 (65.22) | 80 (49.69) |  | 172 (29.91) | - | - |  | 89 (15.48) | 35372932 |
| 36 | Spain | Hospitalized | 1096 | (13.4) | 548 (50) | 44 (8.03) | 211 (38.5) |  | 270 (24.64) | - | - |  | 52 (4.74) | 34079618 |
| 37 | USA | Hospitalized | 1545 | 442 (28.61) | 608 (39.35) | 259 (42.6) | 354 (58.22) |  | 198 (12.82) | - | - |  | 26 (1.68) | 35280117 |
| 38 | USA | ICU | 4221 | - | 2361 (55.93) | 2361 (100) | 588 (24.9) |  | - | - | - |  | 876 (20.75) | 34871701 |
| 39 | USA | Hospitalized | 153 | 19 (12.42) | 62 (40.52) | - | - |  | 33 (21.57) | - | - |  | - | 34710516 |
| 40 | USA | Hospitalized | 1074 | - | 641 (59.68) | - | - |  | - | - | - |  | - | 35356906 |

Note: AKI, acute kidney injury; CKD, chronic kidney disease; ICU, intensive care unit.

**
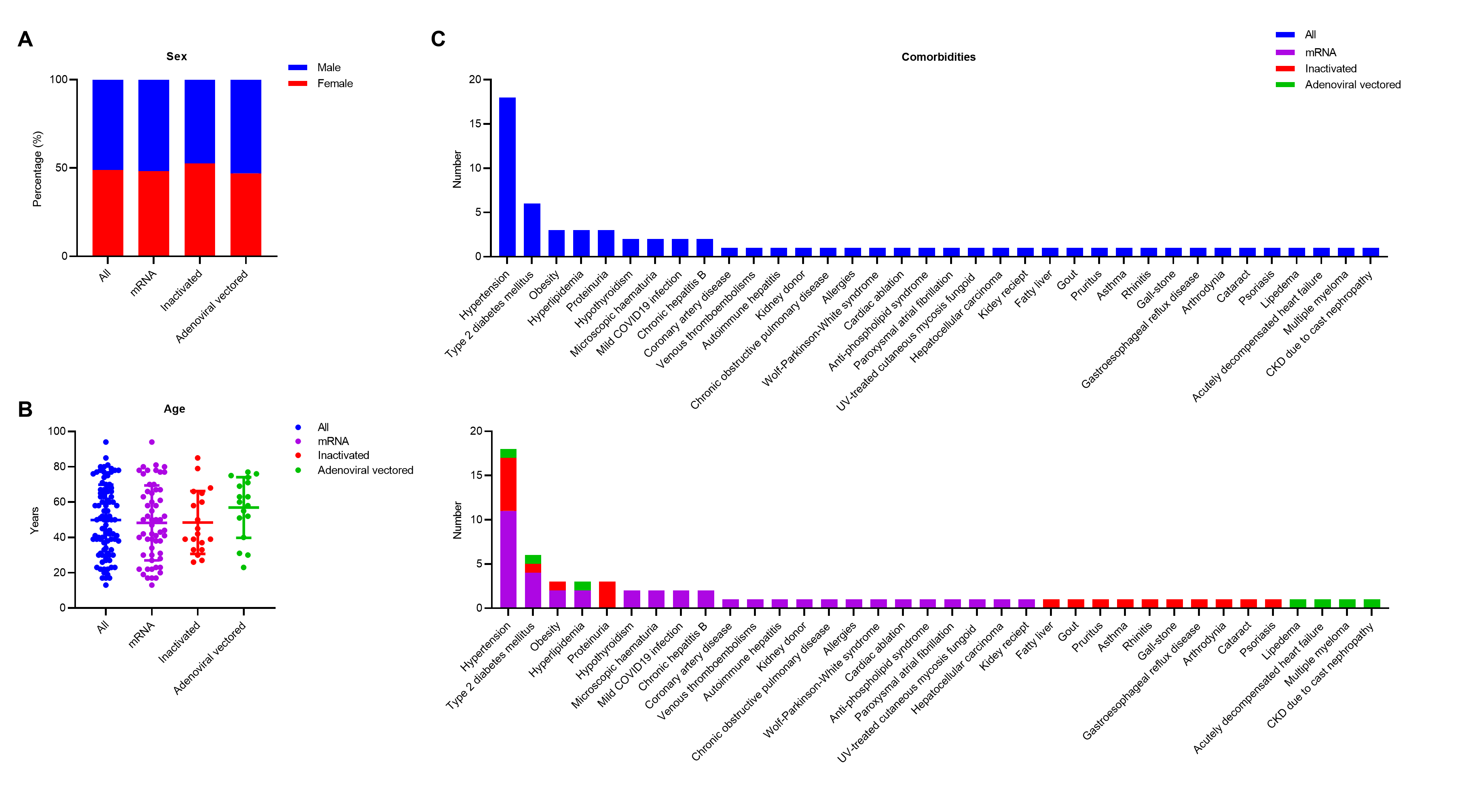
**

**Supplementary Figure 1. Baseline information of kidney diseases after receiving different types of SARS-CoV-2 vaccine.**


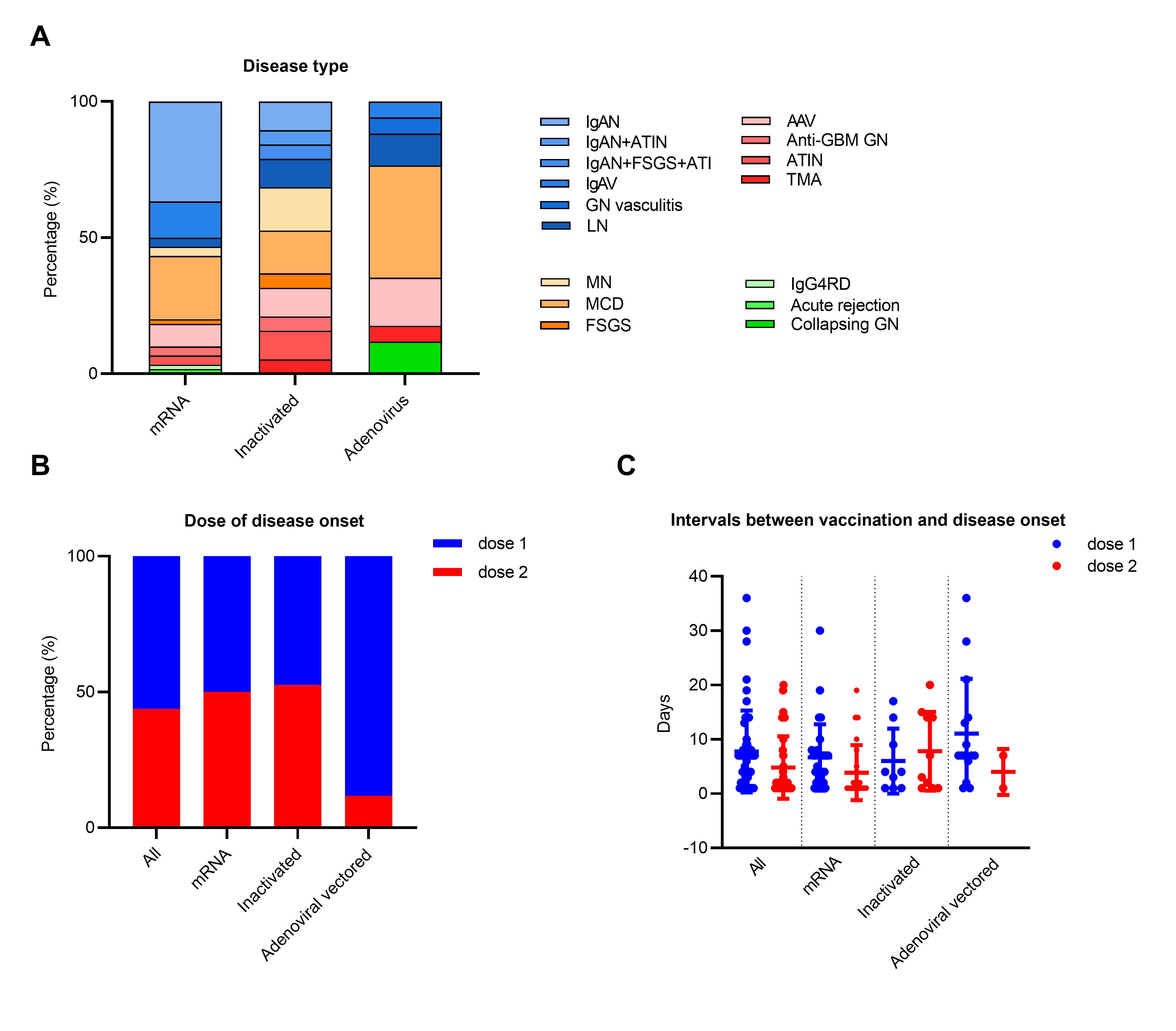


**Supplementary Figure 2. Spectrum and timeline of kidney diseases after receiving different types of SARS-CoV-2 vaccine.**


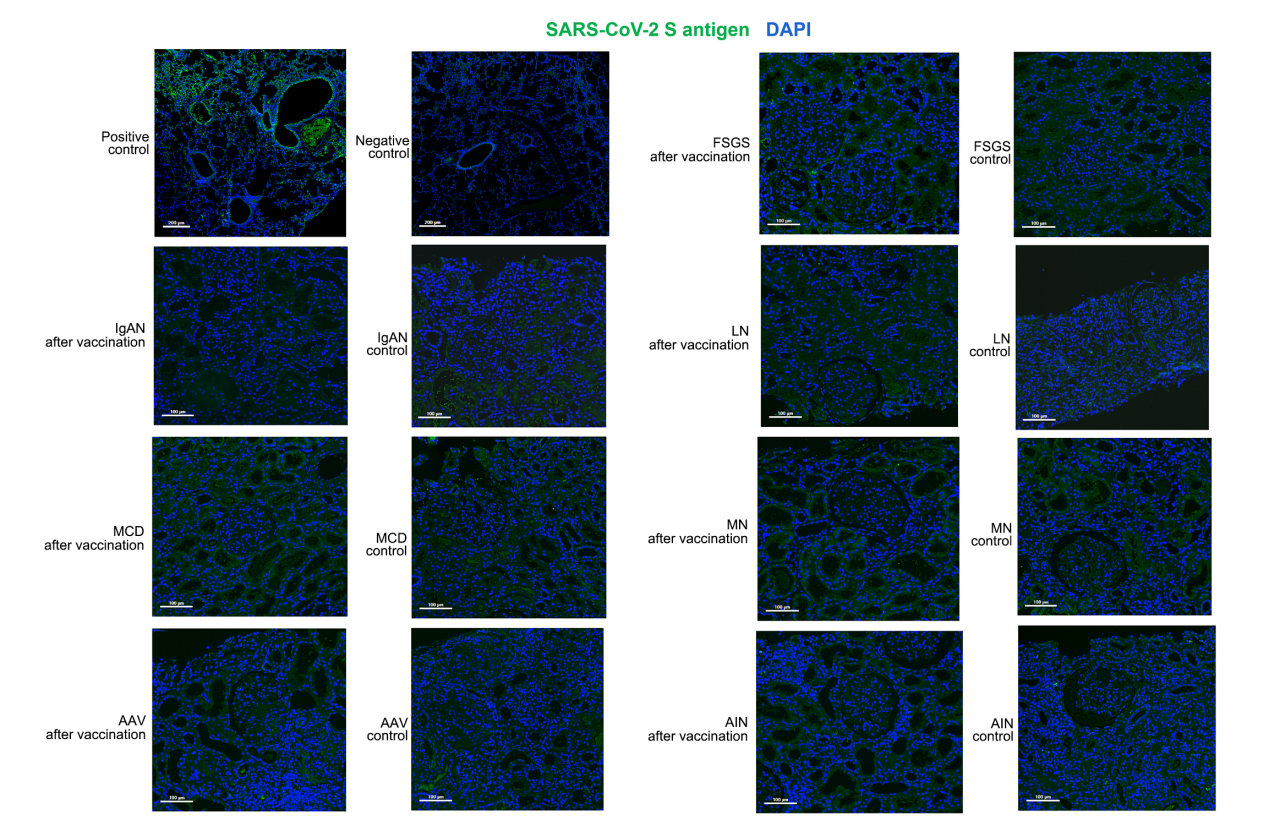


**Supplementary Figure 3. Immunostaining of SARS-CoV-2 spike protein in paraffin-embedded kidney tissues from kidney disease patients after inactivated SARS-CoV-2 vaccination and pathology-matched controls enrolled in 2019.**


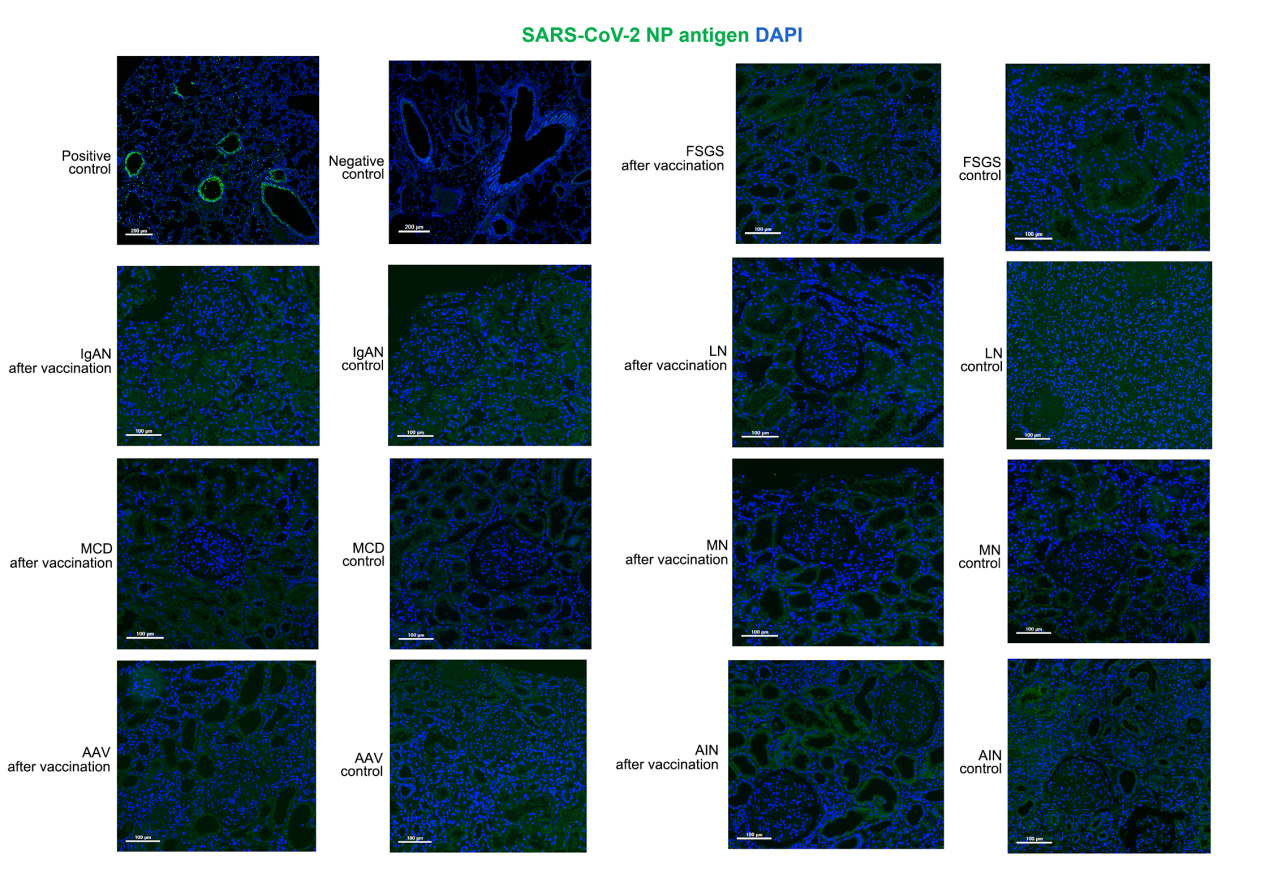


**Supplementary Figure 4. Immunostaining of SARS-CoV-2 nucleoprotein in paraffin-embedded kidney tissues from kidney disease patients after inactivated SARS-CoV-2 vaccination and pathology-matched controls enrolled in 2019.**


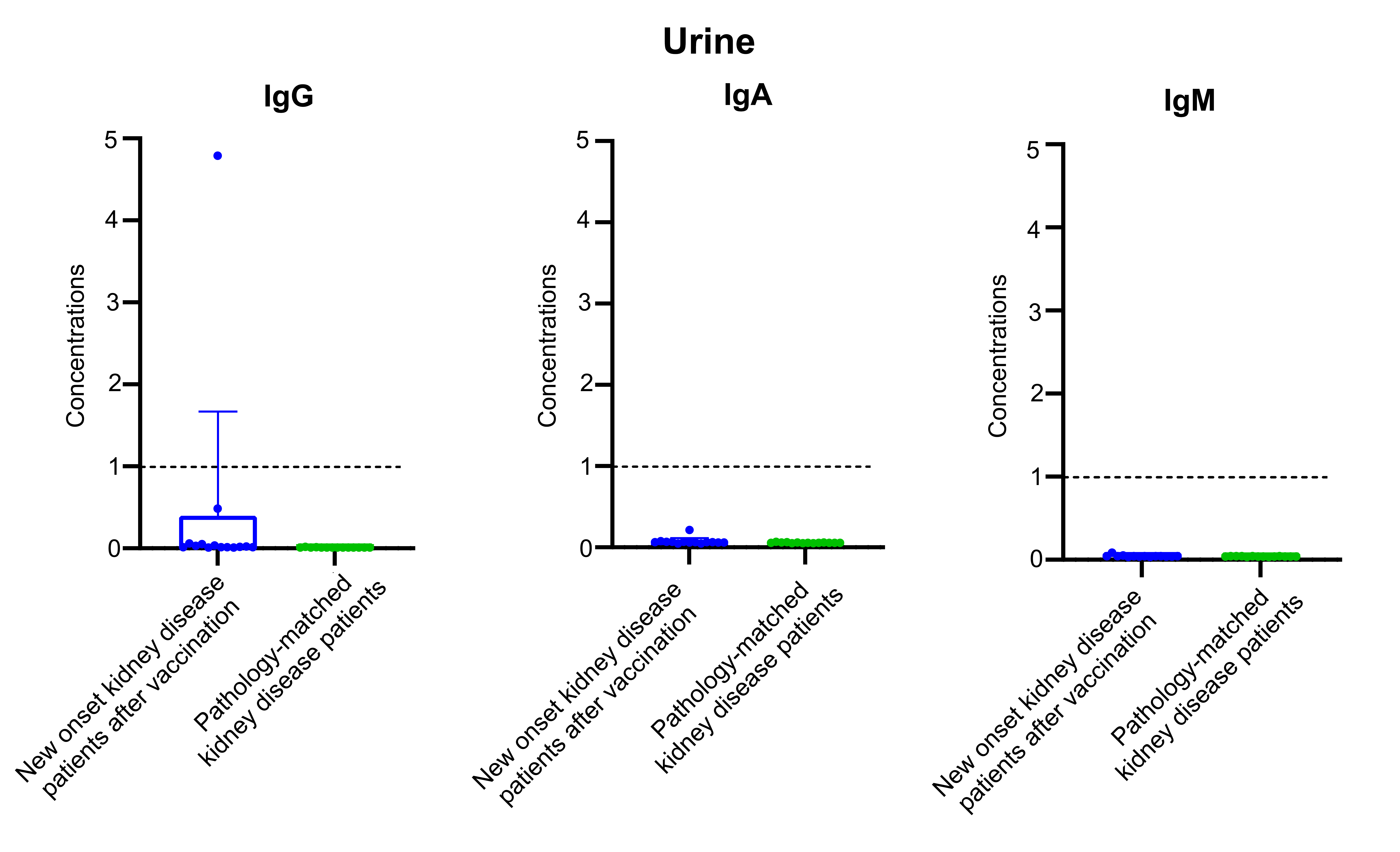


**Supplementary Figure 5. Analyses of SARS-CoV-2-specific antibodies in urine samples of kidney disease patients after inactivated SARS-CoV-2 vaccination and pathology-matched controls enrolled in 2019.**


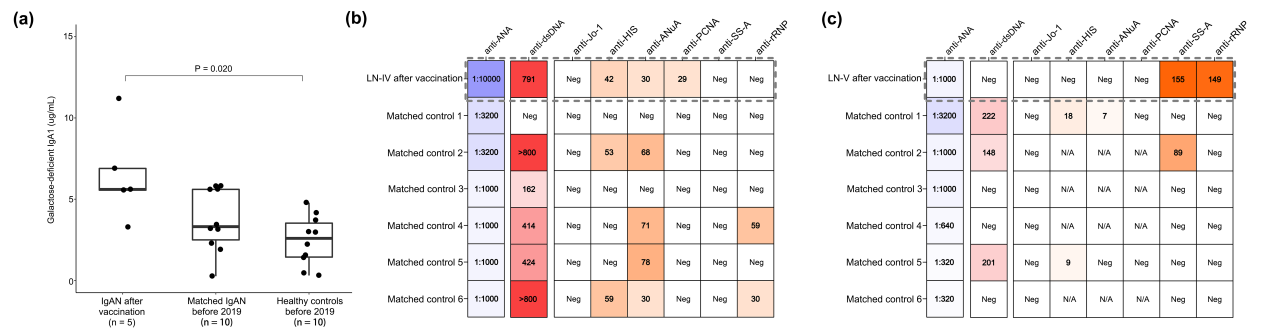


**Supplementary Figure 6. Comparison of pathogenic antibodies in IgA nephropathy and lupus nephritis after inactivated SARS-CoV-2 vaccination with matched controls.**
